# Clinical advantage of targeted sequencing for unbiased tumor mutational burden estimation in samples with low tumor purity

**DOI:** 10.1136/jitc-2020-001199

**Published:** 2020-10-19

**Authors:** Tae Hee Hong, Hongui Cha, Joon Ho Shim, Boram Lee, Jongsuk Chung, Chung Lee, Nayoung K D Kim, Yoon-La Choi, Soohyun Hwang, Yoomi Lee, Sehhoon Park, Hyun Ae Jung, Ji-Yeon Kim, Yeon Hee Park, Jong-Mu Sun, Jin Seok Ahn, Myung-Ju Ahn, Keunchil Park, Se-Hoon Lee, Woong-Yang Park

**Affiliations:** 1 Samsung Genome Institute, Samsung Medical Center, Seoul, Korea; 2 Department of Digital Health, Samsung Advanced Institute of Health Science and Technology, Sungkyunkwan University, Seoul, Korea; 3 Division of Hematology-Oncology, Department of Medicine, Samsung Medical Center, Sungkyunkwan University School of Medicine, Seoul, Korea; 4 Department of Health Science and Technology, Samsung Advanced Institute of Health Science and Technology, Sungkyunkwan University, Seoul, Korea; 5 GENINUS Inc, Seoul, Korea; 6 Department of Pathology and Translational Medicine, Samsung Medical Center, Sungkyunkwan University School of Medicine, Seoul, Korea; 7 Department of Molecular Cell Biology, Sungkyunkwan University School of Medicine, Suwon, Korea

**Keywords:** immunotherapy, biomarkers, tumor, computational biology

## Abstract

**Background:**

Tumor mutational burden (TMB) measurement is limited by low tumor purity of samples, which can influence prediction of the immunotherapy response, particularly when using whole-exome sequencing-based TMB (wTMB). This issue could be overcome by targeted panel sequencing-based TMB (pTMB) with higher depth of coverage, which remains unexplored.

**Methods:**

We comprehensively reanalyzed four public datasets of immune checkpoint inhibitor (ICI)-treated cohorts (adopting pTMB or wTMB) to test each biomarker’s predictive ability for low purity samples (cut-off: 30%). For validation, paired genomic profiling with the same tumor specimens was performed to directly compare wTMB and pTMB in patients with breast cancer (paired-BRCA, n=165) and ICI-treated patients with advanced non-small-cell lung cancer (paired-NSCLC, n=156).

**Results:**

Low tumor purity was common (range 30%–45%) in real-world samples from ICI-treated patients. In the survival analyzes of public cohorts, wTMB could not predict the clinical benefit of immunotherapy when tumor purity was low (log-rank p=0.874), whereas pTMB could effectively stratify the survival outcome (log-rank p=0.020). In the paired-BRCA and paired-NSCLC cohorts, pTMB was less affected by tumor purity, with significantly more somatic variants identified at low allele frequency (p<0.001). We found that wTMB was significantly underestimated in low purity samples with a large proportion of clonal variants undetected by whole-exome sequencing. Interestingly, pTMB more accurately predicted progression-free survival (PFS) after immunotherapy than wTMB owing to its superior performance in the low tumor purity subgroup (p=0.054 vs p=0.358). Multivariate analysis revealed pTMB (p=0.016), but not wTMB (p=0.32), as an independent predictor of PFS even in low-purity samples. The net reclassification index using pTMB was 21.7% in the low-purity subgroup (p=0.016).

**Conclusions:**

Our data suggest that TMB characterization with targeted deep sequencing might have potential strength in predicting ICI responsiveness due to its enhanced sensitivity for hard-to-detect variants at low-allele fraction. Therefore, pTMB could act as an invaluable biomarker in the setting of both clinical trials and practice outside of trials based on its reliable performance in mitigating the purity-related bias.

## Background

Immune checkpoint inhibitors (ICIs) have ushered in a new era of clinical oncology. The tumor mutational burden (TMB), devised as a surrogate for the neoantigen load, is increasingly being accepted as a relevant biomarker for predicting the response to ICI therapy.^1–3^ Nevertheless, the lack of harmonization across various next-generation sequencing (NGS) platforms and the limited predictive performance of tissue-based TMB estimates remain major barriers for precision immunogenetic approaches.^4–6^

Real-world clinical samples are inevitably and frequently associated with a limitation of low tumor content (ie, low purity).^7^ Anagnostou *et al* recently revealed the confounding impact of tumor purity on TMB estimates, emphasizing the need for an integrated biomarker that exhibits improved correlation with outcomes.^8^ This purity-related bias reduces TMB estimates in samples with low tumor purity, thereby increasing the risk of false-negative prediction for the response to ICIs. To effectively predict the response to ICIs on the basis of TMB, there is an urgent need to identify the causes and clinical consequences of the bias due to tumor purity.

Although considered the gold standard, whole-exome sequencing (WES)-based TMB (wTMB) estimation is not currently feasible or expedient in clinical settings.^9^ In theory, the ‘gold standard’ should be an accurate measurement of the total mutations that are capable of being recognized by the immune system as foreign antigens. With its limited depth of coverage (ranged 100 x-200x), WES might not have sufficient sensitivity for detecting variants at low-allele fraction, and might not be able to capture some of the mutations in real-world clinical samples with low tumor purity. In contrast, with the growing use of targeted NGS panels, more and more clinical trials have begun adopting panel-based TMB estimates (pTMB) as a stratification biomarker.^4 10^ The higher depth of coverage achieved by targeted NGS allows for the detection of variants at lower allelic fractions, enabling more sensitive detection of clonal variants in low-purity samples.^8^ Our premise is that measuring TMB with a small fraction of the exome with high depth of coverage using targeted deep sequencing (pTMB) can result in a better estimate of the true TMB than measuring TMB with the entire exome with low coverage (wTMB), particularly for clinical samples with low tumor purity. To evaluate this hypothesis, we reanalyzed the data of four publicly available ICI-treated cohort datasets. In addition, we examined an internal cohort profiled by paired NGS (WES and panel sequencing) to compare the clinical value of pTMB and wTMB.

## Methods

### Study design and cohort characteristics

**Figure 1:**
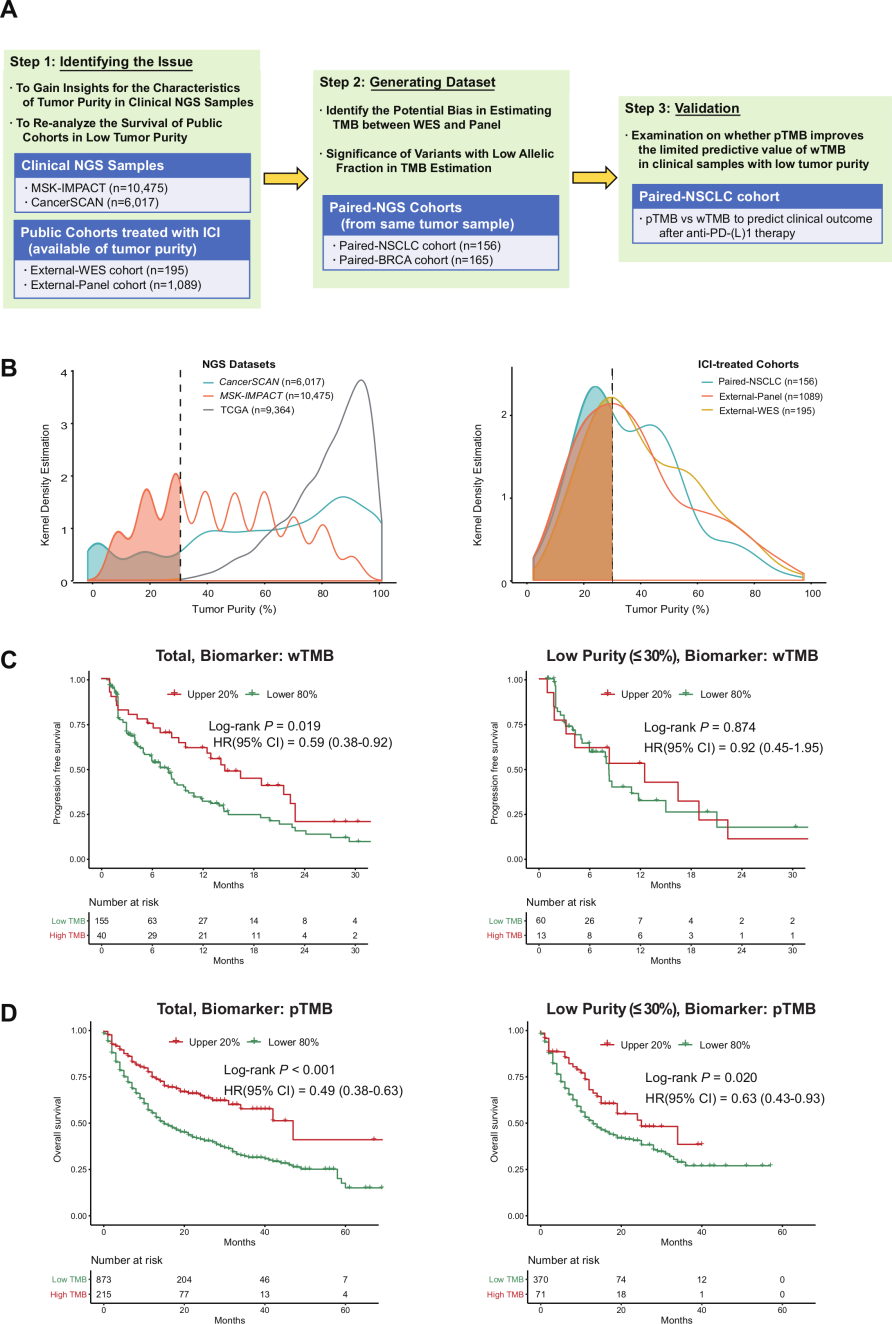
Study design, the distribution of tumor purity and reanalysis of public cohorts. (A) Study overview and cohort characteristics. (B) Distribution of tumor purity from two NGS datasets (CancerSCAN, MSK-IMPACT) and clinical cohorts treated with ICI (external-WES cohort, external-panel cohort and paired-NSCLC cohort of this study). (C) Influence of tumor purity on predictive performance of WES-based TMB in the External-WES cohort (n=195). (D) Influence of tumor purity on predictive performance of panel-based TMB in the external-panel cohort (n=1089). BRCA, breast cancer; ICI, immune checkpoint inhibitor; NGS, next-generation sequencing; NSCLC, non-small-cell lung cancer; pTMB, panel sequencing-based tumor mutational burden; SMC, Samsung Medical Center; TCGA, The Cancer Genome Atlas; TMB, tumor mutational burden; WES, whole-exome sequencing; wTMB, whole-exome sequencing-based tumor mutational burden.

This study was divided into three separate analyzes (figure 1A), including identification of the effects of tumor purity in the predictive performance of TMB from public data, generation of paired-NGS cohorts and clinical validation. The distribution of the tumor purity estimates across clinical NGS samples was investigated in two datasets of a targeted sequencing panel, CancerSCAN^11^ (n=6017) and Memorial Sloan Kettering-Integrated Mutation Profiling of Actionable Cancer Targets^12^ (MSK-IMPACT, n=10 475), which was compared with that of The Cancer Genome Atlas (TCGA) samples^13^ (n=9364). In addition, survival data of public cohorts^1 8 9 14^ treated with immunotherapy were re-analyzed with respect to the efficacy of the two biomarkers in the context of low tumor purity. A total of four public cohorts were classified based on the type of biomarker (wTMB or pTMB) used. The ‘External-WES’ cohort comprised 195 ICI-treated patients with non-small-cell lung cancer (NSCLC) profiled with WES from three cohorts,^8 9 14^ and the ‘External-Panel’ cohort comprised 1089 ICI-treated patients profiled with panel sequencing.^1^ Additional information on data acquisition is described in online supplemental data 1.

10.1136/jitc-2020-001199.supp1 Supplementary data

From June 2014 to April 2019, a total of 279 NSCLC patients treated with antiprogrammed death-1/programmed death ligand-1 (PD-L1) agents were profiled with WES^15^ at our institution. Among them, 156 patients with available remaining material were additionally profiled with CancerSCAN (‘paired-NSCLC cohort’) for this analysis. The same tumor sample was used for both panel sequencing and WES in all (100%) patients; 144 (92.3%) were from the same DNA aliquot. In addition, a dataset containing a total of 165 patients with breast cancer with paired NGS data (‘paired-BRCA cohort’) was also generated in the same manner.^16^ Detailed information of the patient cohorts is presented in online supplemental table S1 and S2.

10.1136/jitc-2020-001199.supp2 Supplementary data

Patients in the paired-NSCLC cohort with data for the response to ICI treatment and survival were further analyzed to validate the clinical value of pTMB in parallel with wTMB according to tumor purity. All patients provided informed consent for sample acquisition for research purposes.

### Assessment of response to ICI treatment

For patients that received ICI treatment, the objective response was assessed by physicians using the Response Evaluation Criteria in Solid Tumors V.1.1. Patients showing a complete response or partial response were defined as responders, whereas patients with stable disease or progressive disease were classified as non-responders. The objective response rate (ORR) was defined as the proportion of responders. Progression-free survival (PFS) was calculated from the start date of ICI treatment to the date of progression or death.

### NGS workflow

Targeted NGS was performed using CancerSCAN V.2, a tumor-only, targeted NGS platform designed at Samsung Medical Center with exonic regions of 381 cancer-related genes (1.07 Mb) and intronic regions of 23 genes in which fusion frequently occurs.^11^ DNA extraction, library preparation and raw data generation for WES and targeted NGS were conducted as previously described.^11 17^ Somatic variants and germline variants were detected by MuTect2^18^ and the GATK HaplotypeCaller,^19^ respectively. Sequencing errors and false-positive variants were manually curated using Integrative Genomics Viewer. The mean sequencing coverages of WES across all tumor samples and blood samples were 160× and 107×, respectively. The mean coverage of targeted NGS across all tumor samples was 829×. Details of the NGS workflow and variant detection are described in online supplemental data 1.

TMB and tumor purity estimation wTMB (expressed as mutations) was estimated as the total number of somatic non-synonymous single nucleotide variants (SNVs) and indels. The limit of detection (LOD) for wTMB estimation was set to a variant allele frequency (VAF) of 5%. The pTMB (expressed as mutations/Mb) was estimated as the total number of somatic non-synonymous SNVs and indels divided by the covered coding region. The LOD for pTMB estimation was set to a VAF of 1%. The subtraction of germline variants for targeted NGS was performed using the available germline databases,^20–24^ and its performance was assessed in the paired NGS dataset by comparing two types of pTMB (tumor-only vs tumor/matched normal, online supplemental figure S1). To evaluate the appropriateness of the panel size, correlations between the pairs of pTMB and wTMB were calculated by in silico simulations of stepwise alterations in the number of included genes (online supplemental figure S2).

10.1136/jitc-2020-001199.supp3 Supplementary data

To control for the effect of interobserver variability on pathological determination of tumor purity, this analysis adopted computational purity metrics. In detail, tumor purity was determined on the basis of four computational algorithms: FACETS,^25^ Sequenza,^26^ PureCN^27^ and a mutation-based estimation using the median VAF. Tumor purity was primarily inferred from FACETS according to the online manual. The other algorithms were prioritized based on their ability to reliably impute missing or unreliable values from FACETS (online supplemental figure S3A). For the analysis of targeted NGS data without WES, PureCN was implemented as the primary algorithm for tumor purity estimation. Manual curation of the results from each algorithm (eg, allele-specific copy number and ploidy levels) was performed in all cases, and details for determining tumor purity are presented in online supplemental figure S3B. For analyzes of external cohorts, TMB and tumor purity data were retrieved from the original publications.^1 8 9 14^

### PD-L1 expression and tumor-infiltrating lymphocytes analysis

Immunohistochemistry of PD-L1 was performed using the Dako PD-L1 IHC 22C3 pharmDx kit (Agilent Technologies, Santa Clara, California, USA). PD-L1 expression scores were reported as the proportion of stained tumor cells, as determined by the thoracic pathologists. PD-L1 subgroups were stratified based on low (0%–49%) and high (≥50%) expression.

Tumor-infiltrating lymphocyte (TIL) was assessed under H&E sections by two independent thoracic pathologists to control the confounding effect of TIL on outcomes. It was reported as continuous variable (proportion score) according to the current pathological guideline.^28^

### Cut-off points and statistical analysis

The samples were stratified into two groups based on the TMB (high vs low) using the top 20% as the cut-off point.^1^ The cut-off point for tumor purity was set to 30%, based on the degree of the purity-related bias in TMB estimates (see online supplemental data 1 and online supplemental figure S4). Details of statistical analysis are described in online supplemental data 1.

## Results

### Characteristics of tumor purity and pTMB in real-world datasets

All analyzed ICI-treated cohorts, as well as the two targeted NGS datasets (CancerSCAN and MSK-IMPACT), showed even distributions of tumor purity, whereas TCGA samples exhibited a skewed distribution with an overrepresentation of high tumor purity (figure 1B). Samples with low tumor purity accounted for 30.4% of all analyzed samples (4993 of 16 551 samples) across the two real-world NGS datasets, whereas the TCGA cohort rarely contained samples with low purity (8 out of 9364 included samples, 0.1%; p<0.001). In ICI-treated cohorts, patients with tumor samples of low purity accounted for 40.5% of all patients (583 of 1440 patients; range, 30.3%–44.9%).

The distribution of pTMB from the two datasets was similar across cancer types and was consistent with previous results for cancer types with high and low mutation rates^29^ (online supplemental figure S5A). However, the pTMB for CancerSCAN was higher than that for MSK-IMPACT (median difference, 3.1 mutations/Mb). The germline components that passed the common single nucleotide polymorphism filter (median, 2.9 mutations/Mb) in the tumor-only pipeline largely explained the observed difference in pTMB between the two datasets (online supplemental figure S5B).

### Reanalyses of survival outcomes in public cohorts treated with ICI

For the External-WES cohort (n=195), wTMB-based survival outcome stratification was effective (log-rank p=0.019, left side of figure 1C). However, wTMB failed to predict PFS in the low-purity subgroup (log-rank p=0.874, right side of figure 1C). For the external-panel cohort (n=1089), the predictive performance of pTMB was high (log-rank p<0.001, left side of figure 1D), even for the low-purity subgroup (log-rank p=0.020, right side of figure 1D).

### Comparative analysis of the effect of tumor purity on pTMB and wTMB estimates

**Figure 2:**
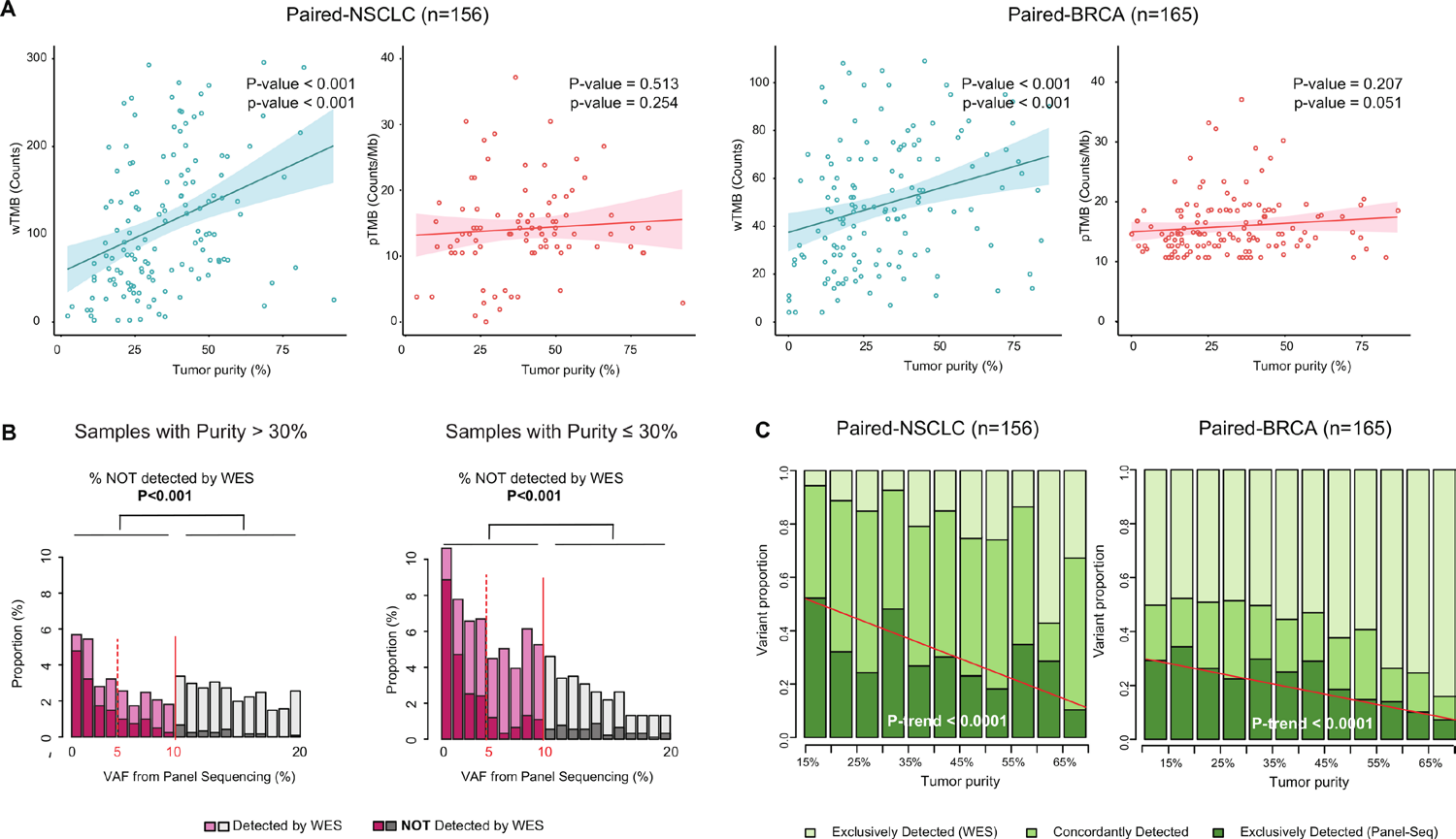
Differential impacts of tumor purity on the two biomarkers. (A) Impact of tumor purity on two types of biomarkers identified from the linear regression. Left: paired-NSCLC cohort; right: paired-BRCA cohort. (B) Prevalence of variants with a low allele fraction. Red lines at 5% and 10% indicate common cut-off points for VAF in pTMB estimation. (C) Trend in the configuration of platforms (by which variants were detected) under changes of tumor purity. BRCA, breast cancer; NSCLC, non-small-cell lung cancer; pTMB, panel sequencing-based tumor mutational burden; VAF, variant allele frequency; WES, whole-exome sequencing; wTMB, whole-exome sequencing-based tumor mutational burden.

Using paired NGS data (ie, pTMB and wTMB for the same tumor specimen), we evaluated the effect of tumor purity on TMB estimates and their correlation. A linear regression analysis showed that tumor purity significantly influences wTMB (p<0.001) but not pTMB (p=0.513, left side of figure 2A). These results were reproducible in the paired-BRCA cohort (right side of figure 2A).

In the paired-NSCLC cohort, there was substantial disagreement in TMB groups between the two platforms (positive percent agreement (PPA)=64.5%, online supplemental table S3). This discrepancy was more prominent in the low-purity subgroup (PPA for adequate vs low-purity subgroups, 68.5% vs 58.3%), suggesting that there is a serious risk of misclassification in samples with low purity.

### More sensitive detection of low-VAF variants in panel sequencing

**Figure 3:**
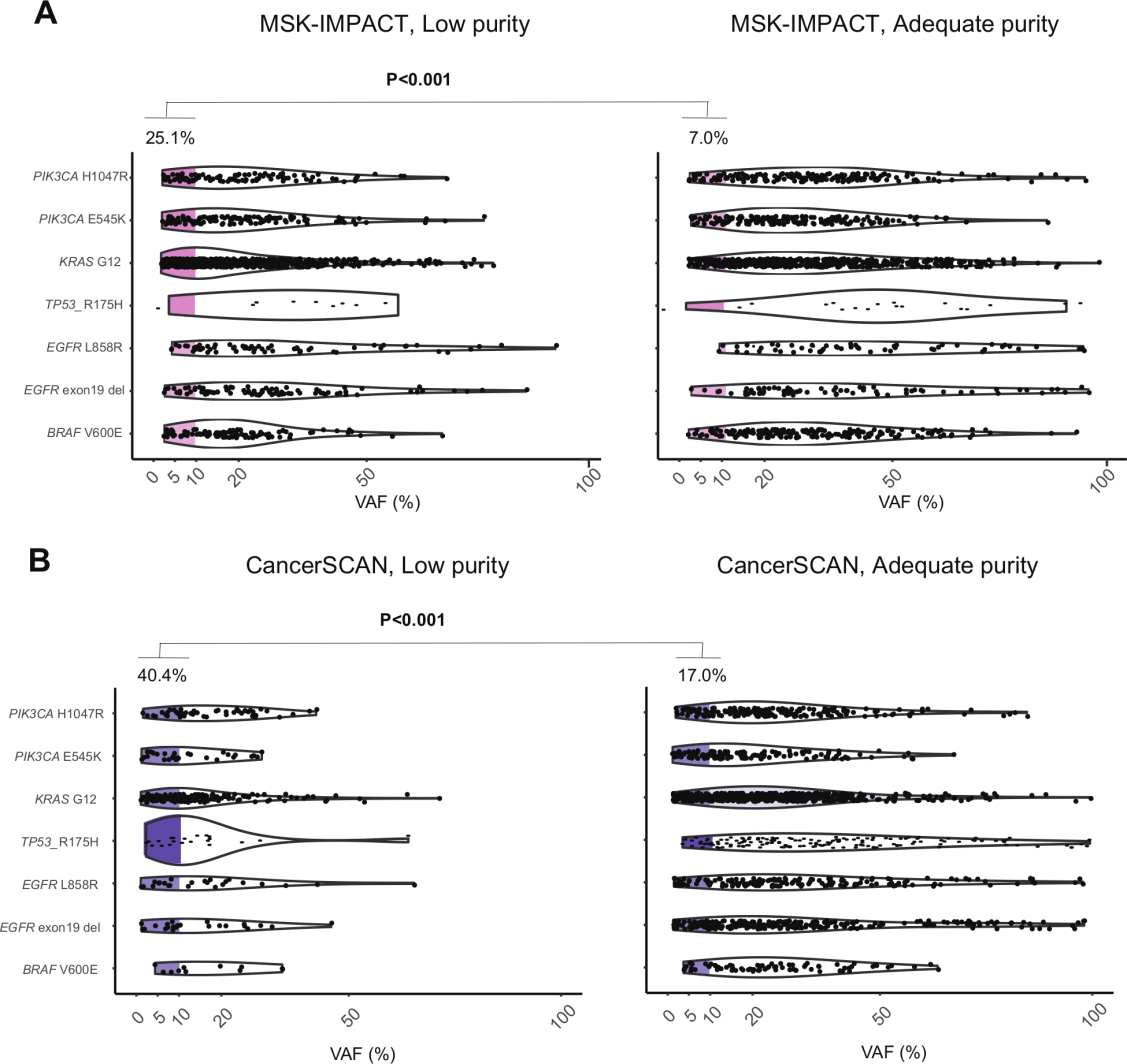
Prevalence of SNVs and Indels with low VAF according to the tumor purity groups from the two NGS datasets. (A) MSK-IMPACT (n=10 475). (B) CancerSCAN (n=6017). Left, adequate purity (>30%) samples, right: low-purity samples. NGS, next-generation sequencing; SNV, single-nucleotide variant; VAF, variant allele frequency.

Next, we investigated mechanistic factors related to the difference in the susceptibility of each platform with respect to tumor purity. In the paired cohort of NSCLC and BRCA, the somatic variants detected by panel sequencing were characterized and visualized based on their VAF (figure 2B). The percentages of variants not detected by WES was much higher in the low-VAF region (p<0.001). More importantly, the proportion of low-VAF variants not detected by WES was increased in low-purity samples compared with that in high-purity samples (23% vs 15%, p<0.001). To explore the influence of tumor purity in the VAF of clonal variants, the VAF distribution of hotspot SNVs was analyzed in 16 492 clinical samples across the two targeted NGS datasets, CancerSCAN and MSK-IMPACT (figure 3). Compared with high-purity samples, the low-purity samples presented a larger proportion of hotspot variants with low VAF less than 10% (40.4% vs 17.0%, p<0.001, figure 3B). This result suggests that low-VAF variants, which are primarily detected by panel sequencing, can arise not only from subclonal variants but also from clonal variants in low-purity samples.

To confirm the higher sensitivity of targeted NGS as compared with WES, somatic variants of the paired-NSCLC cohort were classified on the basis of the NGS platform on which they were detected. Astoundingly, the proportion of variants detected exclusively by targeted NGS tended to increase as tumor purity decreased (p trend <0.001, left side of figure 2C). This trend was reproducible in the paired-BRCA cohort (p trend <0.001, right side of figure 2C).

### Superior clinical performance of pTMB at predicting the benefits of anti-PD-(L)1 therapy in samples with low tumor purity

**Figure 4:**
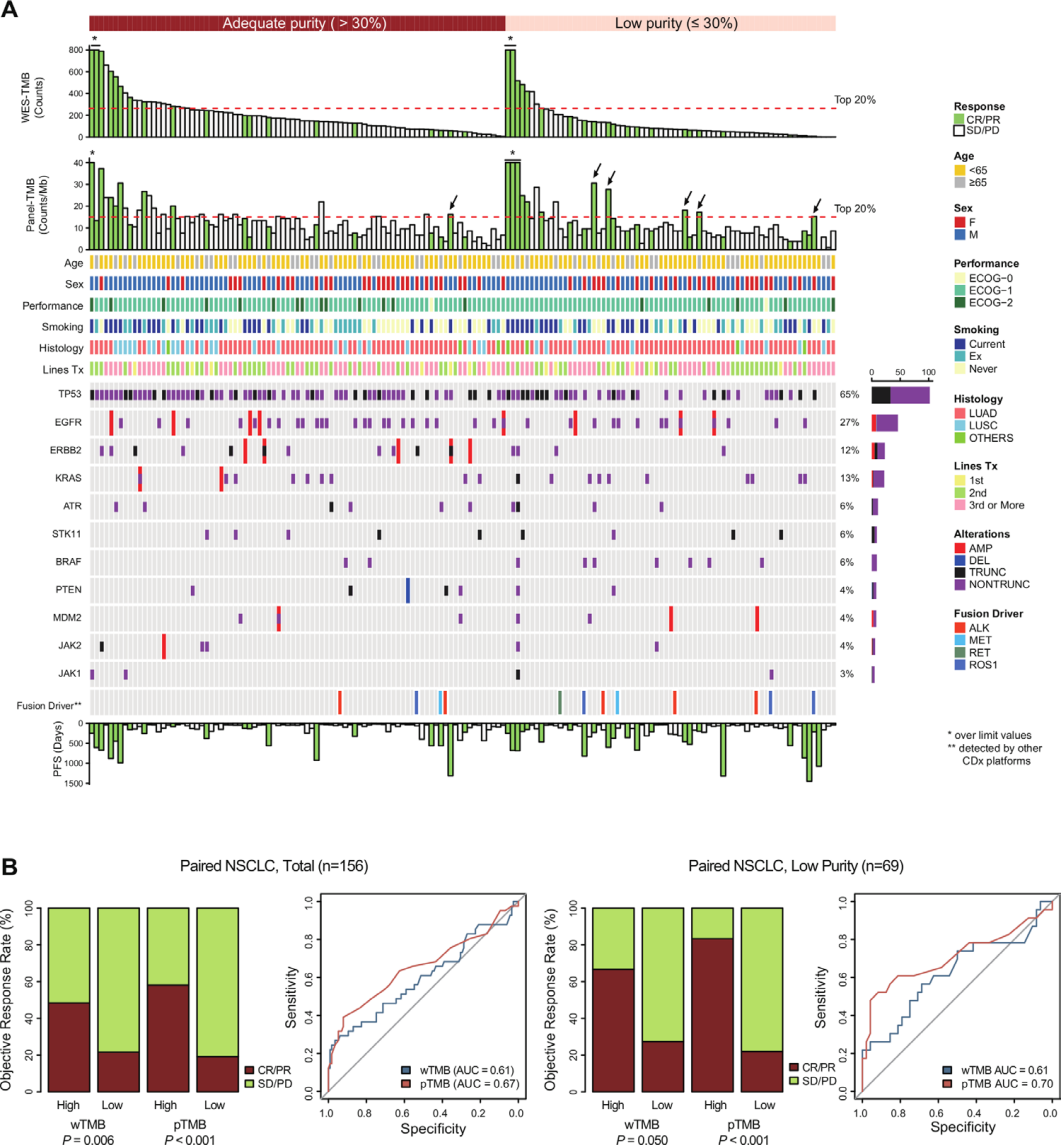
Clinical and genomic characteristics of the paired-NSCLC cohort and responder prediction using the two biomarkers. (A) Heatmap illustrating the clinical and genomic data of 156 patients in the paired-NSCLC cohort. Arrows indicate correctly reclassified patients using pTMB. (B) Comparison of the two biomarkers (wTMB vs pTMB) in the objective response rate and ROC curve analysis (left side: total patients, right side: patients with low purity). AUC, area under the curve; CDx, companion diagnostics; ECOG, Eastern Cooperative Oncology Group; NSCLC, non-small-cell lung cancer; LUAD, lung adenocarcinoma; LUSC, lung squamous cell carcinoma; PFS, progression-free survival; pTMB, panel sequencing-based tumor mutational burden; ROC, receiver operating characteristic; WES, whole-exome sequencing; wTMB, whole-exome sequencing-based tumor mutational burden.

The paired-NSCLC cohort was further scrutinized to study the association between purity-related bias on TMB and impaired prediction for the ICI response. Figure 3A summarizes the clinicogenomic characteristics for 156 patients. Patients correctly reclassified using pTMB were enriched in the low-purity subgroup (black arrows, figure 4A).

Regarding the ORR, both wTMB and pTMB effectively discriminated responders from non-responders among all paired-NSCLC patients (wTMB: 48.4% vs 21.6%, p=0.006; pTMB: 58.1% vs 19.2%, p<0.001, figure 4B). In the subgroup analysis of the low-purity group (n=69), wTMB could not sufficiently discriminate responders with borderline significance (66.7% vs 27.4%, p=0.050), whereas pTMB had superior predictive ability (83.3% vs 22.0%, p<0.001, figure 4B). pTMB had higher accuracy than wTMB for predicting the response (area under the curve of 0.61 for wTMB and 0.70 for pTMB). The net reclassification in the low-purity subgroup was significantly improved on using pTMB instead of wTMB (categorical net classification improvement: 21.7%, 95% CI 4.1% to 39.4%, p=0.016). The performance of pTMB as a stratification biomarker was also superior than wTMB when the effects of immune cell infiltration is controlled (online supplemental figure S6).

**Figure 5:**
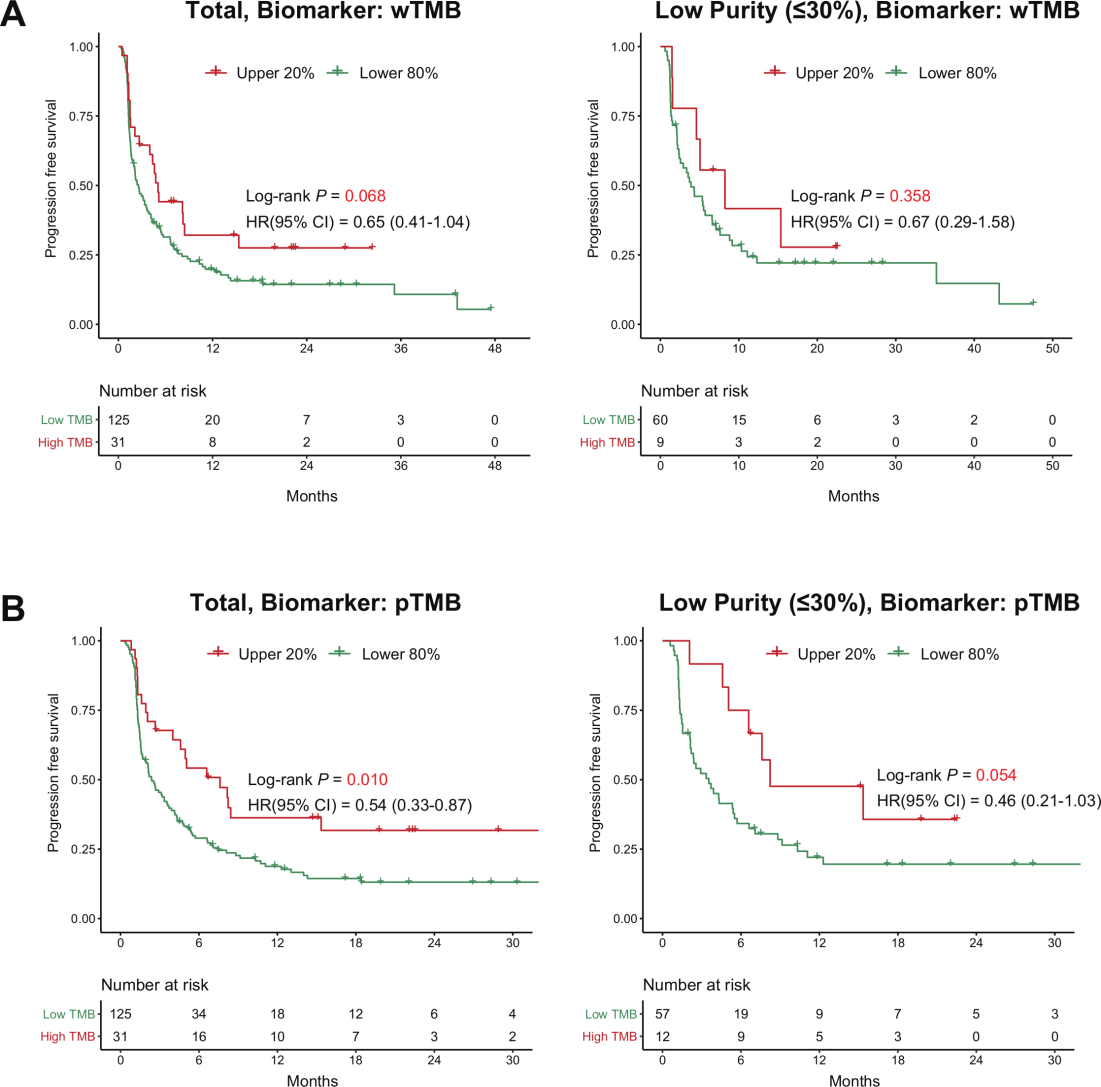
Survival analysis of the paired-NSCLC cohort treated with ICI using the two biomarkers. (A) The use of wTMB as a stratification biomarker (left side: total patients, right side: patients with low-purity samples). (B) The use of pTMB as a stratification biomarker. (Left side: total patients, right side: patients with low-purity samples). ICI, immune checkpoint inhibitor; pTMB, panel sequencing-based tumor mutational burden; wTMB, whole-exome sequencing-based tumor mutational burden.

In terms of survival outcome, a high wTMB tended to stratify patients based on PFS with borderline significance (p=0.068), but could not predict PFS in the subgroup with low purity (HR 0.67, p=0.358, figure 5A). Interestingly, a high pTMB not only predicted longer PFS in all patients (median PFS, 7.61 months vs 2.39 months, HR 0.54, p=0.010) but also provided improved prediction for PFS in the subgroup with low purity (median PFS, 8.23 months vs 3.54 months, HR 0.46, p=0.054, figure 5B). In terms of overall survival (OS), the use of pTMB tends to work better in the OS stratification compared with the use of wTMB though it lacked statistical significance (online supplemental figure S7). After adjusting for age, sex, performance status, and lines of treatment, pTMB was consistently identified as an independent predictor of PFS across all purity groups (low-purity group; adjusted HR 0.32, 95% CI 0.21 to 1.41, p=0.016, table 1). In contrast, the predictive value of wTMB in the multivariate model was substantially reduced in the low-purity subgroup (adjusted HR 0.54, 95% CI 0.21 to 1.41, p=0.212, table 1). Sensitivity analysis including PD-L1 expression as a covariate demonstrated the superiority of pTMB over wTMB for predicting the ICI response in the low-purity subgroup (online supplemental table S4).

**Table 1:** Multivariate Cox regression analyzes for PFS of the paired-NSCLC cohort stratified by tumor purity

Variables		Total patients (n=156)				Adequate purity (n=87)				Low purity (n=69)			
		Model 1 (WES-TMB)		Model 2 (panel-TMB)		Model 1 (WES-TMB)		Model 2 (panel-TMB)		Model 1 (WES-TMB)		Model 2 (panel-TMB)	
		aHR (95% CI)	P value	aHR (95% CI)	P value	aHR (95% CI)	P value	aHR (95% CI)	P value	aHR (95% CI)	P value	aHR (95% CI)	P value
Age	(cont)	1 (0.99 to 1.02)	0.762	1 (0.98 to 1.02)	0.923	0.99 (0.97 to 1.01)	0.335	0.99 (0.96 to 1.01)	0.257	1.01 (0.98 to 1.04)	0.583	1.02 (0.99 to 1.05)	0.313
Sex	M	1 (Reference)	–	1 (Reference)	–	1 (Reference)	–	1 (Reference)	–	1 (Reference)	–	1 (Reference)	–
	F	1.07 (0.72 to 1.59)	0.725	1.08 (0.73 to 1.58)	0.705	1.21 (0.72 to 2.04)	0.467	1.29 (0.77 to 2.15)	0.329	0.88 (0.46 to 1.7)	0.707	0.77 (0.40 to 1.48)	0.436
Lines of therapy received	0	1 (Reference)	–	1 (Reference)	–	1 (Reference)	–	1 (Reference)	–	1 (Reference)	–	1 (Reference)	–
	1	1.64 (0.81 to 3.3)	0.166	1.63 (0.81 to 3.28)	0.17	2.04 (0.76 to 5.51)	0.157	1.86 (0.7 to 4.98)	0.214	1.27 (0.43 to 3.72)	0.666	1.61 (0.54 to 4.80)	0.391
	2	1.11 (0.53 to 2.32)	0.777	1.12 (0.53 to 2.34)	0.766	2.67 (0.95 to 7.53)	0.064	2.78 (0.99 to 7.85)	0.053	0.58 (0.19 to 1.79)	0.345	0.65 (0.21 to 1.98)	0.227
	3	1.37 (0.65 to 2.87)	0.407	1.37 (0.65 to 2.88)	0.408	1.81 (0.66 to 4.98)	0.249	1.59 (0.58 to 4.36)	0.37	0.94 (0.29 to 3.06)	0.914	1.33 (0.40 to 4.49)	0.641
ECOG PS	0	1 (Reference)	–	1 (Reference)	–	1 (Reference)	–	1 (Reference)	–	1 (Reference)	–	1 (Reference)	–
	1	2.22 (0.3 to 16.21)	0.431	2.35 (0.32 to 17.17)	0.399	ND	0.996	ND	0.996	0.41 (0.05 to 3.4)	0.408	0.39 (0.05 to 3.27)	0.383
	2	3.9 (0.5 to 30.58)	0.195	4.18 (0.53 to 32.8)	0.174	ND	0.996	ND	0.996	1.34 (0.13 to 13.39)	0.802	1.15 (0.12 to 11.53)	0.902
**TMB group**	Low	1 (Reference)	–	1 (Reference)	–	1 (Reference)	–	1 (Reference)	–	1 (Reference)	–	1 (Reference)	–
	**High**	**0.59** **(0.36 to 0.97**)	**0.037**	**0.5** **(0.3 to 0.82**)	**0.006**	**0.49** **(0.26 to 0.90**)	**0.022**	**0.46** **(0.24 to 0.89**)	**0.02**	**0.54** **(0.21 to 1.41**)	**0.212**	**0.32** **(0.13 to 0.82**)	**0.016**

aHR, adjusted HR; ECOG, Eastern Cooperative Oncology Group; ND, not determined; NSCLC, non-small-cell lung cancer; PFS, progression-free survival; PS, performance status; TMB, tumor mutational burden; WES, whole-exome sequencing.

To demonstrate the robustness of our analysis in the setting of different thresholds for tumor purity and TMB, we performed several sensitivity analyzes. In accordance with previous results, pTMB tended to act as a better stratification biomarker than wTMB in the setting of different definitions for low tumor purity (<25% and <35%, online supplemental figure S8). In addition, when TMB was considered as a continuous variable rather than selecting the top 20% as a cut-off, we observed that pTMB was consistently identified as an independent predictor of PFS across all purity groups (low-purity group; adjusted HR 0.75, 95% CI 0.58 to 0.96, p=0.021) while wTMB was not (low-purity group; adjusted HR 0.96, 95% CI 0.82 to 1.10, p=0.572; Online supplemental figure S9).

## Discussion

To our knowledge, this study represents the largest series of WES-targeted NGS paired datasets analyzed to date to determine whether targeted NGS could more accurately estimate TMB, even for a high proportion of samples with low tumor purity. Several studies^9 30^ have indicated the feasibility of pTMB to predict clinical benefits from immunotherapy with good correlations against wTMB; however, there has been no investigation on whether pTMB overcomes the limitation of wTMB for predictions with low-purity samples. Minimizing the impact of tumor purity on TMB estimates has implications for the interpretation of trial results and for individual patient management outside of clinical trials.^31^

Our analysis is grounded on the importance of low-purity samples in real-world clinical settings^11^ and the increased sensitivity of targeted NGS for scant variant detection. To address this complex issue, we used a three-step approach: (1) reanalysis of survival in public ICI-treated cohorts with real-world characteristics of tumor purity in clinical samples evaluated by NGS, (2) analysis of the relative susceptibility of each biomarker to purity-related bias and its mechanistic explanation and (3) comparative analysis of the predictive value of wTMB and pTMB for low-purity samples.

The observed consistency of pTMB distributions for two datasets (CancerSCAN and MSK-IMPACT) could reflect the reliability of both targeted NGS platforms. Several factors impact the reliability of pTMB, including the size^32 33^ (whether the panel covers a sufficient area of the genome), correlation (between pTMB and wTMB), and the filtering algorithms used for germline variants.^34 35^ We controlled for the aforementioned factors to ensure the validity of pTMB assessed by CancerSCAN. Although harmonization across various targeted NGS panels is still required, evidence for the validity of targeted NGS-based TMB estimation across various tumor types is accumulating.^6 9 12 30 36^ Of note, the QuIP study^6^ recently compared seven commercial targeted NGS panels and demonstrated the general reliability of pTMB estimates, regardless of panel type.

In particular, the impact of germline variants on TMB estimates appears to be non-negligible. WES always uses matched germline analysis and is free of this issue; however, as we demonstrated, wTMB has weak points in low tumor purity settings due to the limited depth of coverage, which might pose a bigger problem. As for panel sequencing, both tumor-only (eg, Foundation, CancerSCAN) and matched germline analysis (eg, MSK-IMPACT) can be used. In NGS panels adopting a tumor-only approach, unfiltered germline variants can affect the TMB estimates. In these cases, the absolute value may be falsely overestimated, resulting in a slight increase in the overall TMB. However, applying a rank-based cut-off point, along with harmonization across other panel-based platforms, would alleviate this bias. In addition, it is urgent to develop/apply an algorithm that can more thoroughly remove germline variants in tumor-only sequencing methods.

Our preliminary analysis also sheds light on the real-world distribution of tumor purity in clinical samples profiled by targeted NGS. It is well known that TCGA excludes >50% of all samples submitted based on tumor purity (<60%).^7 13^ However, it is noteworthy that samples with low purity (<30%) accounted for a significant portion of the real-world samples from both large NGS datasets and ICI-treated cohorts. Accordingly, the unsatisfactory performance of wTMB in patients with low tumor purity is a serious hurdle for the future application of TMB in precision medicine.

We observed that targeted NGS is highly sensitive for the detection of variants at low allelic fractions. According to a previous study,^11^ usual depth of coverage in WES (150 x-200x) showed a sensitivity of only about 40% for variants with 5% VAF; however, panel sequencing offers depth of coverage typically ranging around 800 x or more, which can detect more than 99% of low-VAF (<5%) variants. Taken together, our results might imply the clinical need for deep sequencing with a lower LOD for accurate TMB estimation. Nevertheless, further studies are required to determine the ideal LOD (eg, VAF under 5% or 1%) for estimating pTMB considering the risk of including false-positive reads arising from deamination artifacts and subclonal variants with uncertain immunogenicity, as well as the need for the sensitive detection of clonal variants in low-purity samples. Our result also has an implication for expanding the limited evaluability in TMB estimation, which remains a major concern for implementing tissue-based TMB as a biomarker for patient selection. Even when the sample’s purity used for TMB analysis is low (<10%–20%), patients who might have been excluded from the trials adopting wTMB can be evaluated for accurate TMB estimation with greater sensitivity of a targeted NGS panel.

Ultimately, our main aim was to assess whether the predictive power of wTMB in clinical samples with low tumor purity can be improved using pTMB. The uniqueness of paired NGS data along with ICI responses enabled us to comprehensively compare TMB estimates from each NGS platform. We can reasonably judge that purity-related misprediction is infrequent in previous and ongoing clinical trials using pTMB.

Our data also challenge conventional notions about TMB. To be specific, WES-based TMB estimates should be interpreted with caution when there are many samples with low purity (<30%), suggesting that WES should be reconsidered as the gold standard. Despite reflection of the total neoantigen burden by definition, the reliability of wTMB as a biomarker in low-purity samples is uncertain owing to its limited ability to effectively call somatic variants with low frequency. At the same time, our study emphasizes the accuracy of targeted NGS, especially for clinicians who have long-standing doubts regarding this approach. Considering other advantages of panel sequencing (eg, reduced cost, expanded tissue availability and shorter turn-around time), our data provide important insight into the robustness of pTMB for clinical use, and may facilitate the use of pTMB as a relevant biomarker for the immunotherapy response.

Our study had the following limitations. First, our survival analyzes were primarily based on PFS instead of OS. The use of PFS as a surrogate for OS is considered an inherent limitation of small-sized biomarker studies using TMB and should be addressed by large-scale prospective trials. Second, despite including the largest series of cohorts with paired NGS data to date, our analysis still had insufficient statistical power. For a sufficient number of low-purity samples, paired NGS data should be generated for a greater number of patients, which may be unrealistic. Finally, there were 12 (7.7%) NSCLC patients whose DNA aliquot was not identically used for sequencing. However, even in those cases, the DNA was extracted from the same tissue block. Moreover, this bias is unlikely to be large since the same DNA was used in most (>90%) patients.

In conclusion, our study suggests an advantage in the use of panel sequencing-based TMB for low-purity samples. Targeted panel sequencing provides reliable predictive performance for the response to anti-PD-(L)1 therapy by mitigating the bias from low purity. Clinical use of pTMB can be encouraged for both clinical trials and real-world settings which frequently involve samples with low tumor purity. Since our findings were limited by the small number of patients included, further validation of our findings is warranted in larger cohorts.
