# Supplementary material for: Clinical advantage of targeted sequencing for unbiased tumor mutational burden estimation in samples with low tumor purity
Source: J Immunother Cancer. 2020 Oct 19;8(2):e001199. doi: 10.1136/jitc-2020-001199 (PMC7574938; doi:10.1136/jitc-2020-001199)
Supplement: Supplementary data [file jitc-2020-001199supp001.pdf]

Supplemental Data 1

Clinical Advantage of Targeted Sequencing for Unbiased Tumor Mutational Burden Estimation in Samples with Low Tumor Purity

Supplementary Methods

Source of external data

To determine the distribution of tumor purity and compare pTMB with our institutional panel (CancerSCAN), we used data of 10,945 patients profiled with MSK-IMPACT (published in 2018).[1] After excluding patients with missing values of required data, information on type of cancer, tumor purity, and TMB were extracted for this analysis.

To preliminarily investigate the clinical influence of tumor purity on each biomarker, four published studies with available tumor purity and TMB data were identified and re-analyzed: (a) the Rizvi cohort profiled with WES[2] (n = 49), (b) the Anagnostou cohort profiled with WES[3] (n = 89), (c) the Miao cohort profiled with WES[4] (n = 57), and (d) the Samstein cohort profiled with a targeted NGS panel[5] (n = 1089). For the Rizvi cohort, data of NSCLC patients co-profiled with WES were extracted since other patients profiled with only targeted NGS overlapped with those analyzed in the Samstein cohort. For the Anagnostou cohort, the data from all NSCLC patients were used. For the Miao cohort, including patients with multiple types of cancer, we extracted data of the NSCLC patients only to allow for pooled analysis with the Rizvi and Anagnostou cohorts. To our knowledge, the Samstein cohort (n = 1089) is the only panel sequencing-profiled cohort of ICI-treated patients with available TMB and tumor purity data. We extracted data from this cohort for five tumor subtypes (NSCLC, melanoma, head and neck cancer, colorectal cancer, and bladder cancer) in which TMB effectively predicted overall survival according to the original publication.[5] For survival analyses of public cohorts, the same cut-off criteria used for our own cohort were applied, and high-TMB was defined within an individual cohort. All data from external sources were directly retrieved from the online supplementary information.[1–5]

DNA extraction, library preparation, and sequencing

For WES, genomic DNA from the tumor was extracted from fresh tissues using QIAamp DNA mini kit (Qiagen, Valencia, CA, USA) and from formalin-fixed paraffin-embedded (FFPE) tissues using QIAamp DNA FFPE Tissue kit. Genomic DNA from whole blood was extracted using the QIAamp DNA

Blood Maxi kit. Both tumor and blood DNA were enriched for exonic regions using the SureSelect XT Human All Exon V5 kit (5190-6210, Agilent). Sequencing libraries were constructed for an Illumina Hiseq 2500 system (Illumina) and sequenced in 100-bp paired-end mode of the TruSeq Rapid PE Cluster kit and TruSeq Rapid SBS kit (PE-402-4001, Illumina). We aimed for a mean target coverage of 200× for tumors and 100× for paired blood samples.

For targeted panel sequencing (CancerSCAN version 2), genomic DNA from the tumor was extracted and prepared following the same protocol as used for WES. Genomic DNA was sheared using a Covaris S220 ultrasonicator (Covaris, Woburn MA, USA), which was used to construct a library with CancerSCAN probes and SureSelect XT Reagent Kit (HSQ, Agilent Technologies) following the manufacturer's protocol. After checking for library quality, sequencing was performed on a HiSeq 2500 system (Illumina) with 100-bp reads. We aimed for a mean target coverage of 800× for the targeted sequencing analysis.

Variant detection and details of TMB calculation

For WES, sequencing reads were aligned to the hg19 reference genome using BWA-MEM (v.0.7.5). PCR duplicates were removed by Picard (v.1.93). The mapped reads near putative indels were realigned and base quality was recalibrated using GATK v3.1-1. Variants were detected using MuTect2 for SNVs and using Pindel (v0.2.4) for small indels. The numbers of non-synonymous SNVs and small indels (less than 30 bp) were counted for WES-TMB calculation.

For targeted panel sequencing (CancerSCAN), sequenced reads were aligned to the hg19 reference genome using BWA-MEM (v.0.7.5). SAMTOOLS (v0.1.18), Picard (version 1.93, https://broadinstitute.github.io/picard/), and GATK (version 3.1-1) were used for manipulating the aligned sequenced data. Variants were detected using MuTect2 for SNVs and using Pindel (v0.2.4) for small indels. The numbers of non-synonymous SNVs and small indels (less than 30 bp) were counted for panel sequencing-based TMB calculation. In cases with breast cancer, synonymous SNVs were also included in the TMB calculation to improve the comparison with WES-TMB (see Supplemental Data 3, Figure S2). We applied several filtering steps to filter the putative germline variants as previously described[6]: (i) variants with very high VAF (≥97%), except for hotspot mutations; (ii)

variants with population allele frequency >3% in the >400 normal samples in our database (this is important for removing ethnic-specific variants); and (iii) other frequently detected variants that are likely to be alignment artefacts or located in hard-to-sequence regions, as curated by manual review and compiled in our database.

Definition of clonal variants for the proportion analysis on panel sequencing datasets

To examine the significance of clonal variants that can be captured at low allele fraction by panel sequencing, we defined clonal mutations according to the following criteria: (1) Mutations listed in the OncoKB database and whose predicted effect is "oncogenic", (2) Mutations designated as initial drivers or clonal in the previous study by Jamal-Hanjani *et al.*[7], and (3) Mutations not discussed as a resistance mechanism after therapy nor subclonal in the literature. After applying the above criteria, a total of 364 SNVs and Indels, which can be regarded as clonal mutations (**see Supplemental Table S5**), were analyzed using the variant proportion analysis on the panel sequencing datasets described in Figure 3.

Cut-off point analysis for tumor purity (see Supplemental Data 3, Figure S4)

We investigated the optimal cut-off point of tumor purity with the TMB ratio, defined as the ratio of the median TMB of low-purity samples to the median TMB of total samples.

$$\text{TMB}\ ratio = \frac{Median\ TMB\ (under\ cutoff)}{Median\ TMB\ (total)}$$

The TMB ratio (0–1) reflects the degree of TMB underestimation at the designated cut-off point. For example, if the purity cut-off is 1, low-purity samples are defined as less than 100%, and the ratio should be 1. If the purity cut-off is 0.5, low-purity samples are defined as less than 50%, and the value should be the ratio of the median TMB with purity < 50% to the median TMB of all samples. Therefore, a greater deviation from 1 would indicate that the TMB value was more strongly underestimated. We calculated the TMB ratio from 100% to 0% with a stepwise change of 5%. Two curves depicting the underestimation of each TMB estimate were then constructed (**see Supplemental Data 3, Figure S4**,

in which the x-axis is the purity cut-off and the y-axis is the median TMB ratio) along with calculation of their 95% confidence intervals. WES-based TMB began to decrease earlier, even at relatively high purity (50%). However, compared with WES, panel-based TMB was stably maintained at 20–30% without significant underestimation. WES-based TMB was severely underestimated to about half in low-purity (<30%) samples. This observed difference between the TMB ratio from the two methods was statistically significant at the point of 30%, which was therefore defined as the optimal cut-off point. Accordingly, in the clinical samples with purity less than 30%, significant underestimation of TMB occurred preferentially with the WES-based method. However, panel-based TMB remained relatively stable without underestimation.

Statistical analysis

The Spearman rho coefficient (ρ) was applied for non-parametric correlations and the Pearson correlation coefficient (R) was used to assess correlations between continuous variables. The Cochran-Armitage test for trend was performed to analyze an association between a categorical variable and an ordinal variable. Categorical variables were compared using Pearson's $\chi 2$ test or Fisher's exact test. The distribution of tumor purity across various datasets was estimated using the kernel density estimation method. Positive and negative percent agreement (PPA, NPA) and overall rates of agreement (ORA) were calculated to test the classification performance of WES-based TMB against that of panel-based TMB. Survival curves with 95% confidence intervals for PFS were constructed using the Kaplan-Meier method and were compared using the non-parametric log-rank test. Univariate Cox proportional hazards regression analysis was used to determine the impact of individual parameters on PFS. A multivariate Cox proportional hazards model was employed to determine the impact of parameters on PFS after adjusting for several important covariates (age, sex, performance status, and lines of received treatment). To assess the incremental value for responder prediction afforded by the alternative biomarker, the categorical net reclassification improvement (NRI) was calculated.

All reported *P* values are two-sided, and *P* values of <0.05 were considered statistically significant. Statistical analyses and visualization were performed using R software version 3.6.1 (www.r-

project.org).

References

1. Zehir A, Benayed R, Shah RH, et al. Mutational landscape of metastatic cancer revealed from prospective clinical sequencing of 10,000 patients. *Nat Med*. 2017;23(6):703-713. doi:10.1038/nm.4333

2. Rizvi H, Sanchez-Vega F, La K, et al. Molecular determinants of response to anti-programmed cell death (PD)-1 and anti-programmed death-ligand 1 (PD-L1) blockade in patients with non-small-cell lung cancer profiled with targeted next-generation sequencing. *J Clin Oncol*. 2018;36(7):633-641. doi:10.1200/JCO.2017.75.3384

3. Anagnostou V, Niknafs N, Marrone K, et al. Multimodal genomic features predict outcome of immune checkpoint blockade in non-small-cell lung cancer. *Nat Cancer*. 2020;1(1):99-111. doi:10.1038/s43018-019-0008-8

4. Miao D, Margolis CA, Vokes NI, et al. Genomic correlates of response to immune checkpoint blockade in microsatellite-stable solid tumors. *Nat Genet*. 2018;50(9):1271-1281. doi:10.1038/s41588-018-0200-2

5. Samstein RM, Lee CH, Shoushtari AN, et al. Tumor mutational load predicts survival after immunotherapy across multiple cancer types. *Nat Genet*. 2019;51(2):202-206. doi:10.1038/s41588-018-0312-8

6. Shin HT, Choi Y La, Yun JW, et al. Prevalence and detection of low-allele-fraction variants in clinical cancer samples. *Nat Commun*. 2017;8(1):1-10. doi:10.1038/s41467-017-01470-y

7. Jamal-Hanjani M, Wilson GA, McGranahan N, et al. Tracking the Evolution of Non–Small-Cell Lung Cancer. *N Engl J Med*. 2017;376(22):2109-2121. doi:10.1056/NEJMoa1616288
