# Supplementary material for: Clinical advantage of targeted sequencing for unbiased tumor mutational burden estimation in samples with low tumor purity
Source: J Immunother Cancer. 2020 Oct 19;8(2):e001199. doi: 10.1136/jitc-2020-001199 (PMC7574938; doi:10.1136/jitc-2020-001199)
Supplement: Supplementary data [file jitc-2020-001199supp002.pdf]

Supplemental Data 2

Table S1. Summary of clinical characteristics of the paired-NSCLC cohort.

Patient ID	Age (years)	Sex	Stage at ICI initiation	Smoking Status	Histology	ECOG	Timepoint sample obtained	Same Tissue	Same DNA Aliquot	Treatment	Prior Lines of Systemic Therapy	Best Clinical Benefit	PFS	PFS censor	PD-L1 group	Mean Target Coverage (Panel sequencing)	Mean Target Coverage (WES, tumor)	Mean target coverage (WES, normal)
SMC_001	67.9	1	IV	2	LUAD	1	Before ICI Treatment	o	o	anti-PDL1	0	PR	375	1	High	567	212	122
SMC_002	52.5	2	IV	0	LUAD	1	Before ICI Treatment	o	o	anti-PDL1	1	PD	51	1	NA	687	149	89
SMC_003	68.7	1	IV	1	OTHERS	1	Before ICI Treatment	o	o	anti-PD1	2	PD	95	1	NA	967	157	133
SMC_004	81.3	1	IV	2	LUAD	1	Before ICI Treatment	o	o	anti-PDL1	1	PR	687	0	High	244	170	110
SMC_005	42.2	1	IV	0	LUAD	1	Before ICI Treatment	o	o	anti-PDL1	1	PD	81	1	NA	590	178	96
SMC_006	78.2	1	IV	1	LUAD	1	Before ICI Treatment	o	o	anti-PDL1	1	PR	607	0	NA	550	128	113
SMC_007	60.9	1	IV	1	LUSC	1	Before ICI Treatment	o	o	anti-PDL1	0	SD	133	1	NA	1084	181	104
SMC_008	61.1	2	IV	0	LUAD	1	Before ICI Treatment	o	o	anti-PDL1	1	PR	673	0	Low	661	199	116
SMC_009	62.6	1	IV	2	LUAD	1	Before ICI Treatment	o	o	anti-PD1	4	PD	40	1	NA	778	172	116
SMC_010	72.4	1	IV	1	LUSC	1	After ICI Treatment	o	o	anti-PDL1	1	PD	37	1	NA	819	161	99
SMC_011	49.4	1	IV	2	LUAD	1	Before ICI Treatment	o	o	anti-PD1	6	PR	681	0	Low	605	152	98
SMC_012	62.2	2	IV	0	LUAD	1	Before ICI Treatment	o	o	anti-PDL1	1	SD	202	1	NA	891	135	95
SMC_013	53.7	2	IV	0	LUAD	1	Before ICI Treatment	o	o	anti-PDL1	2	PR	232	1	High	942	213	111
SMC_014	59.4	1	IV	1	LUAD	1	Before ICI Treatment	o	o	anti-PD1	1	PD	37	1	Low	1065	197	116
SMC_015	57.3	2	IV	0	LUAD	1	Before ICI Treatment	o	o	anti-PDL1	1	PD	26	1	Low	211	168	124
SMC_016	60.4	1	IV	1	LUSC	1	Before ICI Treatment	o	o	anti-PD1	1	PD	29	1	Low	506	177	112
SMC_017	69.3	2	IV	0	LUAD	1	Before ICI Treatment	o	o	anti-PDL1	1	PD	22	1	Low	491	192	100
SMC_018	52.6	1	IV	1	LUAD	1	Before ICI Treatment	o	o	anti-PDL1	2	PR	1317	1	NA	414	131	95

SMC_019	84.5	2	IV	0	LUAD	1	Before ICI Treatment	o	o	anti-PD1	0	PR	215	0	High	495	187	86
SMC_020	71.1	2	IV	0	LUAD	1	Before ICI Treatment	o	o	anti-PDL1	1	PD	40	1	Low	308	206	96
SMC_021	67.3	1	IV	2	LUAD	1	Before ICI Treatment	o	o	anti-PDL1	2	PR	360	0	Low	562	149	108
SMC_022	68.3	1	IV	2	LUAD	1	After ICI Treatment	o	o	anti-PDL1	1	SD	148	1	NA	765	180	101
SMC_023	46.6	1	IV	1	LUAD	1	Before ICI Treatment	o	o	anti-PD1	1	SD	113	1	High	360	148	105
SMC_024	65.6	1	IV	1	LUSC	1	Before ICI Treatment	o	o	anti-PD1	0	SD	203	1	Low	632	171	104
SMC_025	48.4	2	IV	0	LUAD	1	Before ICI Treatment	o	o	anti-PD1	3	PD	14	1	Low	180	94	107
SMC_026	44.4	1	IV	0	LUAD	1	Before ICI Treatment	o	o	anti-PDL1	1	SD	229	0	Low	493	183	111
SMC_027	52.3	1	IV	0	LUAD	1	Before ICI Treatment	o	o	anti-PDL1	1	PD	47	1	Low	672	181	110
SMC_028	50.5	1	IV	2	LUAD	1	Before ICI Treatment	o	o	anti-PD1	3	PR	461	0	High	957	178	95
SMC_029	51.9	1	IV	2	LUAD	0	Before ICI Treatment	o	o	anti-PDL1	2	PR	562	0	High	742	162	112
SMC_030	55	2	IV	0	LUAD	1	After ICI Treatment	o	x	anti-PDL1	3	SD	210	1	Low	786	170	87
SMC_031	44.4	1	IV	1	LUSC	1	Before ICI Treatment	o	o	anti-PD1	1	PD	40	1	NA	493	183	111
SMC_032	59.2	1	IV	1	LUSC	1	Before ICI Treatment	o	o	anti-PDL1	1	SD	140	1	High	811	70	139
SMC_033	47	2	IV	0	LUSC	0	Before ICI Treatment	o	o	anti-PDL1	1	PD	65	1	High	325	127	119
SMC_034	69.1	1	IV	2	LUAD	1	Before ICI Treatment	o	o	anti-PDL1	0	SD	398	1	NA	719	150	101
SMC_035	57.4	1	IV	1	LUAD	1	Before ICI Treatment	o	o	anti-PD1	1	SD	269	1	High	376	159	102
SMC_036	55	1	IV	2	LUSC	1	Before ICI Treatment	o	o	anti-PD1	1	SD	64	1	Low	786	170	87
SMC_037	62.6	2	IV	0	LUAD	1	Before ICI Treatment	o	o	anti-PD1	2	PR	524	0	High	778	172	116
SMC_038	71.8	2	IV	0	LUAD	1	Before ICI Treatment	o	o	anti-PD1	2	PD	66	1	Low	402	178	95
SMC_039	62.3	1	IV	2	LUSC	1	Before ICI Treatment	o	o	anti-PD1	1	PR	83	0	High	517	172	109
SMC_040	51.7	2	IV	0	LUAD	1	After ICI Treatment	o	o	anti-PD1	1	PD	89	1	High	1086	135	96
SMC_041	67.1	1	IV	2	LUAD	1	Before ICI Treatment	o	o	anti-PD1	1	PD	44	1	High	670	160	93
SMC_042	64.8	1	IV	2	LUAD	1	Before ICI Treatment	o	o	anti-PD1	1	PD	38	1	Low	406	168	93

SMC_043	63.3	1	IV	1	LUSC	1	Before ICI Treatment	o	o	anti-PD1	2	PD	60	1	High	672	132	94
SMC_044	32.6	2	IV	0	LUAD	1	After ICI Treatment	o	o	anti-PD1	1	PD	47	1	High	595	152	100
SMC_045	47	1	IV	1	OTHERS	1	Before ICI Treatment	o	o	anti-PD1	1	PR	864	0	High	325	127	119
SMC_046	70.5	1	IV	1	LUSC	1	Before ICI Treatment	o	o	anti-PD1	2	PD	37	1	Low	458	95	86
SMC_047	70.8	1	IV	2	LUSC	1	Before ICI Treatment	o	o	anti-PD1	0	SD	145	1	High	826	176	109
SMC_048	50.5	1	IV	2	LUSC	1	Before ICI Treatment	o	o	anti-PD1	0	SD	110	1	Low	957	178	95
SMC_049	64.4	1	IV	2	LUSC	1	Before ICI Treatment	o	o	anti-PD1	1	SD	97	1	Low	702	172	102
SMC_050	68.9	1	IV	1	LUSC	2	Before ICI Treatment	o	o	anti-PDL1	1	PD	31	1	Low	405	183	113
SMC_051	59.5	2	IV	2	LUAD	1	Before ICI Treatment	o	o	anti-PDL1	2	PR	559	0	High	797	161	86
SMC_052	74.4	2	IV	0	LUAD	1	Before ICI Treatment	o	o	anti-PD1	1	PR	562	0	High	448	141	102
SMC_053	64.9	1	IV	1	LUAD	1	Before ICI Treatment	o	o	anti-PD1	3	PD	40	1	High	493	105	97
SMC_054	57.3	1	IV	2	LUAD	1	Before ICI Treatment	o	o	anti-PD1	1	PR	468	1	High	211	168	124
SMC_055	62.7	1	IV	2	LUAD	1	Before ICI Treatment	o	o	anti-PD1	1	PD	64	1	Low	607	115	118
SMC_056	54.9	2	IV	0	LUSC	1	Before ICI Treatment	o	o	anti-PDL1	3	PD	49	1	Low	482	143	112
SMC_057	68.6	1	IV	0	LUAD	1	Before ICI Treatment	o	o	anti-PD1	3	PD	67	1	Low	298	194	99
SMC_058	65.4	1	IV	2	LUSC	1	Before ICI Treatment	o	o	anti-PD1	2	SD	163	1	Low	1144	170	102
SMC_059	50.5	1	IV	2	LUAD	2	Before ICI Treatment	o	o	anti-PD1	2	SD	102	1	Low	957	178	95
SMC_060	50.3	1	IV	2	LUSC	1	Before ICI Treatment	o	o	anti-PDL1	3	CR	987	0	NA	938	167	100
SMC_061	50.8	1	IV	2	LUAD	1	Before ICI Treatment	o	o	anti-PD1	0	PR	1311	0	NA	153	101	114
SMC_062	49.3	2	IV	0	LUAD	1	Before ICI Treatment	o	o	anti-PD1	3	SD	78	1	High	259	154	95
SMC_063	72.1	1	IV	1	LUAD	1	Before ICI Treatment	o	o	anti-PD1	3	SD	82	1	High	925	141	97
SMC_064	62.5	1	IV	2	LUSC	1	Before ICI Treatment	o	o	anti-PDL1	2	SD	34	1	High	691	191	111
SMC_065	72.8	1	IV	1	LUAD	1	Before ICI Treatment	o	o	anti-PDL1	7	PD	72	1	Low	473	141	114
SMC_066	50.3	1	IV	2	LUAD	1	Before ICI Treatment	o	o	anti-PD1	2	SD	174	1	Low	938	167	100

SMC_067	50.3	1	IV	2	LUAD	1	Before ICI Treatment	o	o	anti-PDL1	1	PR	138	0	Low	938	167	100
SMC_068	55.9	1	IV	1	LUAD	1	Before ICI Treatment	o	o	anti-PD1	2	PD	37	1	Low	778	167	87
SMC_069	65.7	1	IV	2	LUAD	1	Before ICI Treatment	o	o	anti-PD1	3	PR	561	1	High	581	165	115
SMC_070	45.5	2	IV	0	LUSC	1	Before ICI Treatment	o	o	anti-PDL1	1	PD	36	1	High	392	175	115
SMC_071	63.6	2	IV	0	LUAD	1	Before ICI Treatment	o	o	anti-PDL1	5	PD	22	1	Low	870	163	110
SMC_072	56.8	2	IV	0	LUSC	1	Before ICI Treatment	o	o	anti-PDL1	2	SD	65	1	NA	860	132	103
SMC_073	78.2	1	IV	1	LUAD	1	Before ICI Treatment	o	o	anti-PD1	2	PD	20	1	Low	550	128	113
SMC_074	57.6	2	IV	0	LUAD	1	After ICI Treatment	o	o	anti-PDL1	1	PD	37	1	NA	517	150	98
SMC_075	39.1	1	IV	2	LUAD	2	Before ICI Treatment	o	x	anti-PDL1	6	PD	37	1	Low	271	134	304
SMC_076	80.5	1	IV	1	LUAD	1	Before ICI Treatment	o	o	anti-PD1	1	PD	28	1	High	621	168	96
SMC_077	66.9	1	IV	2	LUSC	1	Before ICI Treatment	o	o	anti-PD1	2	PR	448	0	High	551	185	112
SMC_078	48.1	1	IV	1	LUAD	1	Before ICI Treatment	o	o	anti-PD1	4	SD	314	0	High	298	174	101
SMC_079	61.3	1	IV	2	LUSC	1	Before ICI Treatment	o	o	anti-PD1	1	PD	59	1	High	716	181	134
SMC_080	52.1	1	IV	1	LUAD	1	After ICI Treatment	o	o	anti-PD1	1	SD	108	1	Low	524	196	127
SMC_081	63.9	1	IV	1	LUSC	1	Before ICI Treatment	o	o	anti-PD1	2	SD	102	1	High	393	162	101
SMC_082	64.7	1	IV	1	LUAD	1	Before ICI Treatment	o	o	anti-PD1	2	PR	257	1	Low	852	168	123
SMC_083	43.1	1	IV	2	LUAD	2	Before ICI Treatment	o	o	anti-PD1	1	PR	249	1	Low	775	165	113
SMC_084	58.7	1	IV	2	LUAD	1	Before ICI Treatment	o	o	anti-PD1	2	PR	205	0	High	759	208	133
SMC_085	57.3	1	IV	2	LUAD	1	Before ICI Treatment	o	o	anti-PDL1	1	SD	315	1	Low	211	168	124
SMC_086	69.9	1	IV	2	OTHERS	1	Before ICI Treatment	o	o	anti-PDL1	3	PR	154	1	High	501	181	148
SMC_087	61.4	2	IV	0	LUAD	1	Before ICI Treatment	o	o	anti-PDL1	2	PR	168	1	Low	638	163	114
SMC_088	66.9	2	IV	0	LUAD	1	Before ICI Treatment	o	o	anti-PD1	1	PD	46	1	NA	551	185	112
SMC_089	74.4	2	IV	0	LUAD	1	Before ICI Treatment	o	o	anti-PDL1	3	PR	201	1	NA	448	141	102
SMC_090	51.4	1	IV	1	LUAD	2	Before ICI Treatment	o	o	anti-PD1	2	PD	25	1	High	608	189	128

SMC_091	60	1	IV	2	LUAD	1	Before ICI Treatment	o	o	anti-PDL1	2	SD	38	1	High	455	163	116
SMC_092	62	1	IV	1	LUAD	2	Before ICI Treatment	o	o	anti-PDL1	2	PD	11	1	Low	916	172	99
SMC_093	73	1	IV	1	LUSC	1	Before ICI Treatment	o	o	anti-PD1	1	PD	34	1	High	682	195	126
SMC_094	56.9	1	IV	2	LUAD	1	Before ICI Treatment	o	o	anti-PD1	1	PD	18	1	High	641	188	113
SMC_095	59.3	1	IV	2	LUAD	1	Before ICI Treatment	o	o	anti-PD1	2	PD	73	1	Low	820	186	114
SMC_096	63.2	1	IV	2	LUAD	1	Before ICI Treatment	o	o	anti-PDL1	1	SD	247	1	Low	447	154	106
SMC_097	58.6	1	IV	2	LUSC	1	Before ICI Treatment	o	o	anti-PD1	1	PR	603	0	High	486	183	7
SMC_098	62.1	2	IV	0	LUSC	1	Before ICI Treatment	o	o	anti-PDL1	3	PD	57	1	High	607	196	104
SMC_099	55	1	IV	2	LUAD	1	Before ICI Treatment	o	o	anti-PDL1	4	PD	34	1	Low	786	170	87
SMC_100	58.8	2	IV	0	LUAD	2	Before ICI Treatment	o	o	anti-PDL1	4	PD	34	1	High	573	156	103
SMC_101	58.9	1	IV	2	LUAD	1	Before ICI Treatment	o	o	anti-PDL1	0	SD	160	0	Low	758	219	104
SMC_102	39.6	1	IV	1	LUAD	1	After ICI Treatment	o	o	anti-PDL1	0	SD	132	1	NA	1090	162	117
SMC_103	71.9	2	IV	0	OTHERS	2	Before ICI Treatment	o	o	anti-PD1	1	SD	59	0	Low	898	161	100
SMC_104	55.3	1	IV	0	LUAD	1	Before ICI Treatment	o	o	anti-PD1	1	PD	35	1	Low	672	133	117
SMC_105	52.9	1	IV	0	LUAD	1	Before ICI Treatment	o	o	anti-PD1	1	PD	39	1	Low	438	231	152
SMC_106	60.3	1	IV	2	LUAD	1	After ICI Treatment	o	o	anti-PD1	2	SD	151	1	Low	433	211	128
SMC_107	68.5	1	IV	1	LUAD	1	Before ICI Treatment	o	x	anti-PD1	1	PD	39	1	Low	1067	144	109
SMC_108	37	1	IV	0	LUAD	1	Before ICI Treatment	o	o	anti-PD1	1	PD	38	1	Low	580	168	102
SMC_109	59.3	1	IV	2	LUSC	1	Before ICI Treatment	o	o	anti-PD1	0	PD	47	1	High	820	186	114
SMC_110	56.9	1	IV	0	LUAD	1	Before ICI Treatment	o	o	anti-PDL1	2	PR	1073	1	NA	641	188	113
SMC_111	72.5	1	IV	2	LUAD	1	Before ICI Treatment	o	o	anti-PDL1	0	SD	63	1	NA	541	100	78
SMC_112	59.4	1	IV	2	OTHERS	1	Before ICI Treatment	o	o	anti-PDL1	1	PR	251	1	High	1065	197	116
SMC_113	61.8	2	IV	0	LUAD	1	Before ICI Treatment	o	o	anti-PDL1	5	PR	672	0	Low	1003	182	111
SMC_114	72.4	2	IV	0	LUAD	1	Before ICI Treatment	o	o	anti-PDL1	1	PR	925	0	NA	819	161	99

SMC_115	58.9	1	IV	2	LUAD	1	After ICI Treatment	o	o	anti-PDL1	2	SD	81	1	High	758	219	104
SMC_116	59.4	1	IV	2	LUAD	1	Before ICI Treatment	o	x	anti-PDL1	1	PR	127	1	High	1065	197	116
SMC_117	50.5	1	IV	2	LUAD	2	Before ICI Treatment	o	o	anti-PDL1	3	PR	881	0	Low	957	178	95
SMC_118	58.3	2	IV	0	LUAD	1	Before ICI Treatment	o	o	anti-PD1	5	PD	34	1	Low	593	166	131
SMC_119	73.6	2	IV	0	LUAD	1	Before ICI Treatment	o	o	anti-PD1	3	PD	43	1	Low	434	169	121
SMC_120	49.4	2	IV	0	LUAD	2	Before ICI Treatment	o	o	anti-PD1	5	PR	227	1	Low	605	152	98
SMC_121	29.5	1	IV	2	LUAD	1	Before ICI Treatment	o	x	anti-PD1	9	SD	215	0	Low	903	150	86
SMC_122	50.6	2	IV	0	LUAD	1	After ICI Treatment	o	x	anti-PDL1	3	SD	80	1	Low	857	125	102
SMC_123	64.7	1	IV	1	LUSC	1	Before ICI Treatment	o	o	anti-PDL1	3	PR	157	1	Low	852	168	123
SMC_124	42.7	2	IV	0	LUAD	1	Before ICI Treatment	o	o	anti-PDL1	3	PD	48	1	High	976	184	100
SMC_125	63.4	2	IV	0	OTHERS	1	Before ICI Treatment	o	x	anti-PD1	1	PD	45	1	NA	967	154	118
SMC_126	69.8	2	IV	0	LUAD	1	After ICI Treatment	o	x	anti-PDL1	1	PD	40	1	NA	1086	166	102
SMC_127	53.3	1	IV	1	LUSC	2	Before ICI Treatment	o	o	anti-PD1	5	SD	122	1	High	1018	142	108
SMC_128	36.9	2	IV	0	LUAD	1	Before ICI Treatment	o	x	anti-PDL1	3	PR	216	1	NA	928	148	97
SMC_129	59.8	2	IV	0	LUAD	2	Before ICI Treatment	o	o	anti-PDL1	4	PD	41	1	Low	1024	104	89
SMC_130	47.6	2	IV	0	LUAD	2	Before ICI Treatment	o	o	anti-PDL1	3	PD	32	1	NA	1216	154	93
SMC_131	63.1	1	IV	1	LUAD	1	Before ICI Treatment	o	o	anti-PD1	2	PR	327	1	High	1029	161	111
SMC_132	62.5	2	IV	0	LUSC	1	After ICI Treatment	o	x	anti-PD1	4	PD	42	1	High	691	191	111
SMC_133	63.1	1	IV	1	LUAD	1	Before ICI Treatment	o	o	anti-PD1	7	PD	41	1	High	1029	161	111
SMC_134	73.7	1	IV	1	LUAD	1	Before ICI Treatment	o	o	anti-PDL1	1	PD	36	1	NA	986	136	94
SMC_135	80	1	IV	1	LUAD	2	Before ICI Treatment	o	x	anti-PDL1	3	PR	382	0	Low	1030	158	108
SMC_136	62.5	1	IV	2	LUAD	1	Before ICI Treatment	o	o	anti-PD1	2	PD	45	1	Low	691	191	111
SMC_137	65.4	1	IV	1	LUAD	1	Before ICI Treatment	o	o	anti-PD1	1	SD	126	1	NA	1144	170	102
SMC_138	62.2	2	IV	0	LUAD	2	Before ICI Treatment	o	o	anti-PDL1	3	PD	44	1	Low	891	135	95

SMC_139	60.9	1	IV	1	LUAD	1	Before ICI Treatment	o	o	anti-PDL1	5	PD	36	1	NA	1084	181	104
SMC_140	61.1	2	IV	0	LUAD	2	Before ICI Treatment	o	o	anti-PDL1	5	PD	28	1	NA	661	199	116
SMC_141	59.7	2	IV	0	LUAD	1	Before ICI Treatment	o	o	anti-PDL1	6	SD	165	1	NA	908	117	81
SMC_142	65.8	2	IV	0	LUAD	1	Before ICI Treatment	o	o	anti-PDL1	1	PD	49	1	NA	860	162	99
SMC_143	59.5	2	IV	0	LUAD	2	Before ICI Treatment	o	x	anti-PD1	2	PD	48	1	Low	797	161	86
SMC_144	49.9	1	IV	1	LUAD	1	Before ICI Treatment	o	o	anti-PDL1	2	SD	338	1	Low	1091	165	104
SMC_145	63.7	1	IV	2	LUSC	1	Before ICI Treatment	o	o	anti-PDL1	2	PD	40	1	NA	1031	169	114
SMC_146	69.9	2	IV	0	OTHERS	1	Before ICI Treatment	o	o	anti-PDL1	5	SD	427	1	NA	501	181	148
SMC_147	49.2	2	IV	0	LUAD	1	Before ICI Treatment	o	o	anti-PDL1	5	PD	39	1	NA	672	137	110
SMC_148	67.3	2	IV	2	LUAD	1	Before ICI Treatment	o	o	anti-PDL1	2	PR	821	0	High	562	149	108
SMC_149	44.1	2	IV	0	LUAD	1	Before ICI Treatment	o	o	anti-PD1	1	SD	279	1	NA	562	144	101
SMC_150	55.9	1	IV	2	LUAD	2	After ICI Treatment	o	o	anti-PD1	5	PD	34	1	Low	778	167	87
SMC_151	55.2	1	IV	2	LUAD	1	Before ICI Treatment	o	o	anti-PDL1	2	PR	1449	0	NA	967	162	103
SMC_152	53.1	2	IV	0	LUAD	1	Before ICI Treatment	o	o	anti-PDL1	1	SD	119	1	NA	989	135	90
SMC_153	75.6	1	IV	2	LUSC	1	Before ICI Treatment	o	o	anti-PDL1	0	SD	131	1	NA	1295	162	114
SMC_154	51.7	2	IV	0	LUAD	1	Before ICI Treatment	o	o	anti-PD1	2	PD	45	1	NA	1086	135	96
SMC_155	71.3	1	IV	1	LUAD	2	Before ICI Treatment	o	o	anti-PDL1	4	PD	52	1	NA	950	127	97
SMC_156	45.6	1	IV	0	LUSC	1	Before ICI Treatment	o	o	anti-PD1	4	SD	436	1	Low	670	158	107

ECOG, Eastern Cooperative Oncology Group; *ICI*, immune checkpoint inhibitor; *LUAD*, lung adenocarcinoma; *LUSC*, lung squamous carcinoma; *PFS*, progression-free survival; *TMB*, tumor mutational burden; *WES*, whole-exome sequencing.

Table S2. Summary of tumor sample characteristics of the paired-NSCLC and paired-BRCA cohort.

Patient ID	Tumor Type	Tumor Purity	Tumor Purity Estimation Algorithm	wTMB (Mutations)	pTMB (Mutations/Mb)
SMC_001	NSCLC	0.225	FACETS	119	14.32029
SMC_002	NSCLC	0.445	FACETS	165	14.32029
SMC_003	NSCLC	0.330	PureCN	9	4.773429
SMC_004	NSCLC	0.247	FACETS	517	842.9876
SMC_005	NSCLC	0.315	FACETS	86	6.682801
SMC_006	NSCLC	0.494	FACETS	1047	13.3656
SMC_007	NSCLC	0.400	FACETS	249	12.41092
SMC_008	NSCLC	0.365	FACETS	788	37.23275
SMC_009	NSCLC	0.461	FACETS	661	23.86715
SMC_010	NSCLC	0.493	FACETS	405	19.09372
SMC_011	NSCLC	0.250	FACETS	894	46.77961
SMC_012	NSCLC	0.235	FACETS	43	7.637487
SMC_013	NSCLC	0.200	FACETS	141	30.54995
SMC_014	NSCLC	0.423	FACETS	128	6.682801
SMC_015	NSCLC	0.243	FACETS	42	7.637487
SMC_016	NSCLC	0.420	FACETS	234	10.50154
SMC_017	NSCLC	0.489	FACETS	103	15.27497
SMC_018	NSCLC	0.176	FACETS	50	5.728115
SMC_019	NSCLC	0.223	FACETS	79	8.592173
SMC_020	NSCLC	0.252	FACETS	135	7.637487
SMC_021	NSCLC	0.282	FACETS	97	8.592173
SMC_022	NSCLC	0.406	FACETS	248	15.27497
SMC_023	NSCLC	0.160	Sequenza	206	9.546858
SMC_024	NSCLC	0.448	FACETS	242	11.45623

SMC_025	NSCLC	0.472	FACETS	269	7.637487
SMC_026	NSCLC	0.272	FACETS	59	8.592173
SMC_027	NSCLC	0.372	FACETS	61	3.818743
SMC_028	NSCLC	0.110	Sequenza	69	18.13903
SMC_029	NSCLC	0.407	FACETS	68	7.637487
SMC_030	NSCLC	0.471	FACETS	97	7.637487
SMC_031	NSCLC	0.180	Sequenza	44	7.637487
SMC_032	NSCLC	0.295	FACETS	303	28.64058
SMC_033	NSCLC	0.244	FACETS	34	5.728115
SMC_034	NSCLC	0.531	FACETS	73	6.682801
SMC_035	NSCLC	0.247	FACETS	74	11.45623
SMC_036	NSCLC	0.373	FACETS	265	15.27497
SMC_037	NSCLC	0.160	PureCN	63	5.728115
SMC_038	NSCLC	0.562	FACETS	73	6.682801
SMC_039	NSCLC	0.397	FACETS	282	20.0484
SMC_040	NSCLC	0.230	PureCN	38	10.50154
SMC_041	NSCLC	0.560	FACETS	195	13.3656
SMC_042	NSCLC	0.036	Mutation-based	18	3.818743
SMC_043	NSCLC	0.341	FACETS	137	7.637487
SMC_044	NSCLC	0.210	FACETS	30	9.546858
SMC_045	NSCLC	0.100	Sequenza	14	8.592173
SMC_046	NSCLC	0.187	FACETS	178	13.3656
SMC_047	NSCLC	0.788	FACETS	337	10.50154
SMC_048	NSCLC	0.712	FACETS	46	8.592173
SMC_049	NSCLC	0.344	FACETS	195	13.3656
SMC_050	NSCLC	0.330	FACETS	127	5.728115
SMC_051	NSCLC	0.295	FACETS	18	3.818743
SMC_052	NSCLC	0.439	FACETS	62	5.728115

SMC_053	NSCLC	0.401	FACETS	225	13.3656
SMC_054	NSCLC	0.237	FACETS	264	17.18435
SMC_055	NSCLC	0.110	Sequenza	53	6.682801
SMC_056	NSCLC	0.400	FACETS	29	5.728115
SMC_057	NSCLC	0.164	FACETS	49	10.50154
SMC_058	NSCLC	0.191	FACETS	2	5.728115
SMC_059	NSCLC	0.257	FACETS	25	4.773429
SMC_060	NSCLC	0.481	FACETS	465	30.54995
SMC_061	NSCLC	0.305	FACETS	59	16.22966
SMC_062	NSCLC	0.295	FACETS	53	5.728115
SMC_063	NSCLC	0.685	FACETS	19	9.546858
SMC_064	NSCLC	0.567	FACETS	323	24.82183
SMC_065	NSCLC	0.230	Sequenza	2	0.954686
SMC_066	NSCLC	0.270	Sequenza	63	6.682801
SMC_067	NSCLC	0.542	FACETS	196	5.728115
SMC_068	NSCLC	0.151	FACETS	78	10.50154
SMC_069	NSCLC	0.428	FACETS	179	10.50154
SMC_070	NSCLC	0.549	FACETS	74	9.546858
SMC_071	NSCLC	0.477	FACETS	85	9.546858
SMC_072	NSCLC	0.087	Mutation-based	14	3.818743
SMC_073	NSCLC	0.499	FACETS	104	15.27497
SMC_074	NSCLC	0.453	FACETS	190	13.3656
SMC_075	NSCLC	0.259	FACETS	87	2.864058
SMC_076	NSCLC	0.262	FACETS	34	6.682801
SMC_077	NSCLC	0.553	FACETS	553	20.0484
SMC_078	NSCLC	0.292	FACETS	69	8.592173
SMC_079	NSCLC	0.473	FACETS	246	16.22966
SMC_080	NSCLC	0.104	Mutation-based	28	11.45623

SMC_081	NSCLC	0.349	FACETS	131	13.3656
SMC_082	NSCLC	0.658	FACETS	323	26.7312
SMC_083	NSCLC	0.725	FACETS	1432	57.28115
SMC_084	NSCLC	0.273	FACETS	483	24.82183
SMC_085	NSCLC	0.210	PureCN	85	9.546858
SMC_086	NSCLC	0.228	FACETS	420	21.95777
SMC_087	NSCLC	0.232	FACETS	187	12.41092
SMC_088	NSCLC	0.356	FACETS	165	9.546858
SMC_089	NSCLC	0.188	FACETS	61	17.18435
SMC_090	NSCLC	0.595	FACETS	142	21.95777
SMC_091	NSCLC	0.247	FACETS	244	14.32029
SMC_092	NSCLC	0.540	FACETS	173	7.637487
SMC_093	NSCLC	0.498	FACETS	279	14.32029
SMC_094	NSCLC	0.229	FACETS	105	8.592173
SMC_095	NSCLC	0.255	FACETS	153	6.682801
SMC_096	NSCLC	0.514	FACETS	149	4.773429
SMC_097	NSCLC	0.260	PureCN	133	27.68589
SMC_098	NSCLC	0.404	FACETS	173	15.27497
SMC_099	NSCLC	0.503	FACETS	73	11.45623
SMC_100	NSCLC	0.497	FACETS	142	7.637487
SMC_101	NSCLC	0.312	FACETS	26	1.909372
SMC_102	NSCLC	0.150	PureCN	27	5.728115
SMC_103	NSCLC	0.226	FACETS	46	8.592173
SMC_104	NSCLC	0.495	FACETS	55	7.637487
SMC_105	NSCLC	0.241	FACETS	188	5.728115
SMC_106	NSCLC	0.381	FACETS	230	16.22966
SMC_107	NSCLC	0.238	FACETS	79	9.546858
SMC_108	NSCLC	0.350	FACETS	36	3.818743

SMC_109	NSCLC	0.211	FACETS	258	13.3656
SMC_110	NSCLC	0.266	FACETS	3	0
SMC_111	NSCLC	0.218	FACETS	207	21.95777
SMC_112	NSCLC	0.166	FACETS	4528	66.82801
SMC_113	NSCLC	0.148	Mutation-based	93	11.45623
SMC_114	NSCLC	0.542	FACETS	146	11.45623
SMC_115	NSCLC	0.476	FACETS	335	18.13903
SMC_116	NSCLC	0.379	FACETS	197	14.32029
SMC_117	NSCLC	0.397	FACETS	604	23.86715
SMC_118	NSCLC	0.170	FACETS	75	12.41092
SMC_119	NSCLC	0.807	FACETS	223	14.32029
SMC_120	NSCLC	0.309	FACETS	49	5.728115
SMC_121	NSCLC	0.690	FACETS	316	7.637487
SMC_122	NSCLC	0.350	FACETS	119	6.682801
SMC_123	NSCLC	0.748	FACETS	363	11.45623
SMC_124	NSCLC	0.350	Sequenza	6	6.682801
SMC_125	NSCLC	0.819	FACETS	300	9.546858
SMC_126	NSCLC	0.418	FACETS	138	8.592173
SMC_127	NSCLC	0.684	FACETS	306	16.22966
SMC_128	NSCLC	0.100	Sequenza	7	15.27497
SMC_129	NSCLC	0.110	Sequenza	2	8.592173
SMC_130	NSCLC	0.452	FACETS	93	13.3656
SMC_131	NSCLC	0.604	FACETS	127	7.637487
SMC_132	NSCLC	0.461	FACETS	107	9.546858
SMC_133	NSCLC	0.467	FACETS	134	11.45623
SMC_134	NSCLC	0.110	Sequenza	49	8.592173
SMC_135	NSCLC	0.681	FACETS	243	13.3656
SMC_136	NSCLC	0.235	FACETS	417	14.32029

SMC_137	NSCLC	0.403	FACETS	148	5.728115
SMC_138	NSCLC	0.200	PureCN	58	7.637487
SMC_139	NSCLC	0.753	FACETS	171	14.32029
SMC_140	NSCLC	0.516	FACETS	103	10.50154
SMC_141	NSCLC	0.150	Sequenza	135	9.546858
SMC_142	NSCLC	0.419	FACETS	208	16.22966
SMC_143	NSCLC	0.636	FACETS	207	11.45623
SMC_144	NSCLC	0.167	FACETS	142	9.546858
SMC_145	NSCLC	0.533	FACETS	321	19.09372
SMC_146	NSCLC	0.464	FACETS	51	12.41092
SMC_147	NSCLC	0.533	FACETS	71	16.22966
SMC_148	NSCLC	0.238	FACETS	151	9.546858
SMC_149	NSCLC	0.283	FACETS	71	8.592173
SMC_150	NSCLC	0.404	FACETS	149	9.546858
SMC_151	NSCLC	0.021	Mutation-based	7	6.682801
SMC_152	NSCLC	0.187	FACETS	108	10.50154
SMC_153	NSCLC	0.212	FACETS	94	9.546858
SMC_154	NSCLC	0.791	FACETS	64	10.50154
SMC_155	NSCLC	0.419	FACETS	163	14.32029
SMC_156	NSCLC	0.921	FACETS	26	2.864058
SMC_157	BRCA	0.150	Sequenza	56	10.50154
SMC_158	BRCA	0.430	FACETS	50	17.18435
SMC_159	BRCA	0.000	FACETS	4	8.592173
SMC_160	BRCA	0.000	FACETS	9	14.32029
SMC_161	BRCA	0.035	Mutation-based	32	11.45623
SMC_162	BRCA	0.039	Mutation-based	59	18.13903
SMC_163	BRCA	0.416	FACETS	74	16.22966
SMC_164	BRCA	0.249	FACETS	48	18.13903

SMC_165	BRCA	0.287	FACETS	68	18.13903
SMC_166	BRCA	0.201	FACETS	35	8.592173
SMC_167	BRCA	0.000	FACETS	11	14.32029
SMC_168	BRCA	0.300	Sequenza	19	22.91246
SMC_169	BRCA	0.478	FACETS	83	14.32029
SMC_170	BRCA	0.130	Sequenza	47	10.50154
SMC_171	BRCA	0.283	FACETS	48	12.41092
SMC_172	BRCA	0.373	FACETS	61	10.50154
SMC_173	BRCA	0.200	Sequenza	68	5.728115
SMC_174	BRCA	0.180	Sequenza	16	12.41092
SMC_175	BRCA	0.110	Sequenza	4	13.3656
SMC_176	BRCA	0.140	Sequenza	66	13.3656
SMC_177	BRCA	0.221	FACETS	48	21.95777
SMC_178	BRCA	0.231	FACETS	166	22.91246
SMC_179	BRCA	0.240	PureCN	19	10.50154
SMC_180	BRCA	0.222	FACETS	17	14.32029
SMC_181	BRCA	0.160	Sequenza	51	17.18435
SMC_182	BRCA	0.360	FACETS	137	10.50154
SMC_183	BRCA	0.370	FACETS	29	11.45623
SMC_184	BRCA	0.190	Sequenza	203	26.7312
SMC_185	BRCA	0.160	Sequenza	31	18.13903
SMC_186	BRCA	0.218	FACETS	56	14.32029
SMC_187	BRCA	0.371	FACETS	73	16.22966
SMC_188	BRCA	0.210	Sequenza	49	19.09372
SMC_189	BRCA	0.110	Sequenza	60	13.3656
SMC_190	BRCA	0.382	FACETS	44	13.3656
SMC_191	BRCA	0.319	FACETS	46	14.32029
SMC_192	BRCA	0.493	FACETS	148	29.59526

SMC_193	BRCA	0.140	Sequenza	138	11.45623
SMC_194	BRCA	0.237	FACETS	132	14.32029
SMC_195	BRCA	0.866	FACETS	90	18.13903
SMC_196	BRCA	0.120	Sequenza	35	10.50154
SMC_197	BRCA	0.162	FACETS	9	13.3656
SMC_198	BRCA	0.350	FACETS	47	10.50154
SMC_199	BRCA	0.333	FACETS	99	20.0484
SMC_200	BRCA	0.150	Sequenza	14	14.32029
SMC_201	BRCA	0.471	FACETS	116	12.41092
SMC_202	BRCA	0.120	Sequenza	25	10.50154
SMC_203	BRCA	0.213	FACETS	57	16.22966
SMC_204	BRCA	0.479	FACETS	125	19.09372
SMC_205	BRCA	0.240	FACETS	68	16.22966
SMC_206	BRCA	0.190	PureCN	18	11.45623
SMC_207	BRCA	0.022	Mutation-based	4	12.41092
SMC_208	BRCA	0.461	FACETS	30	18.13903
SMC_209	BRCA	0.170	PureCN	43	10.50154
SMC_210	BRCA	0.130	Sequenza	69	8.592173
SMC_211	BRCA	0.180	Sequenza	41	10.97596
SMC_212	BRCA	0.403	FACETS	152	28.35457
SMC_213	BRCA	0.150	Sequenza	21	14.63462
SMC_214	BRCA	0.396	FACETS	169	13.71996
SMC_215	BRCA	0.180	Sequenza	127	19.20794
SMC_216	BRCA	0.063	Mutation-based	70	15.54928
SMC_217	BRCA	0.280	FACETS	125	6.402646
SMC_218	BRCA	0.744	FACETS	92	13.71996
SMC_219	BRCA	0.395	FACETS	80	13.71996
SMC_220	BRCA	0.180	Sequenza	108	8.231973

SMC_221	BRCA	0.763	FACETS	67	11.89063
SMC_222	BRCA	0.307	FACETS	89	18.29327
SMC_223	BRCA	0.598	FACETS	84	13.71996
SMC_224	BRCA	0.019	Mutation-based	26	16.46395
SMC_225	BRCA	0.170	Sequenza	76	9.546858
SMC_226	BRCA	0.359	FACETS	43	7.637487
SMC_227	BRCA	0.344	FACETS	63	18.13903
SMC_228	BRCA	0.250	Sequenza	130	10.50154
SMC_229	BRCA	0.561	FACETS	119	9.146637
SMC_230	BRCA	0.310	Sequenza	105	10.97596
SMC_231	BRCA	0.150	Sequenza	32	12.41092
SMC_232	BRCA	0.180	Sequenza	31	7.637487
SMC_233	BRCA	0.220	Sequenza	84	8.592173
SMC_234	BRCA	0.658	FACETS	70	6.682801
SMC_235	BRCA	0.383	FACETS	74	11.45623
SMC_236	BRCA	0.434	FACETS	68	17.18435
SMC_237	BRCA	0.180	Sequenza	43	9.546858
SMC_238	BRCA	0.672	FACETS	32	9.546858
SMC_239	BRCA	0.373	FACETS	137	22.86659
SMC_240	BRCA	0.431	FACETS	52	7.637487
SMC_241	BRCA	0.130	Sequenza	52	14.32029
SMC_242	BRCA	0.827	FACETS	55	10.50154
SMC_243	BRCA	0.755	FACETS	113	20.0484
SMC_244	BRCA	0.362	FACETS	43	5.728115
SMC_245	BRCA	0.321	FACETS	47	7.637487
SMC_246	BRCA	0.347	FACETS	75	13.3656
SMC_247	BRCA	0.435	FACETS	55	9.146637
SMC_248	BRCA	0.300	Sequenza	30	13.3656

SMC_249	BRCA	0.709	FACETS	56	9.546858
SMC_250	BRCA	0.775	FACETS	62	7.31731
SMC_251	BRCA	0.801	FACETS	20	7.31731
SMC_252	BRCA	0.284	FACETS	186	14.32029
SMC_253	BRCA	0.626	FACETS	39	6.682801
SMC_254	BRCA	0.722	FACETS	72	10.50154
SMC_255	BRCA	0.383	FACETS	194	19.20794
SMC_256	BRCA	0.170	Sequenza	28	15.27497
SMC_257	BRCA	0.290	PureCN	24	13.3656
SMC_258	BRCA	0.538	FACETS	95	12.41092
SMC_259	BRCA	0.150	Sequenza	28	18.13903
SMC_260	BRCA	0.310	Sequenza	205	21.00309
SMC_261	BRCA	0.357	FACETS	274	36.27806
SMC_262	BRCA	0.409	FACETS	56	13.3656
SMC_263	BRCA	0.337	FACETS	7	8.592173
SMC_264	BRCA	0.140	Sequenza	46	22.91246
SMC_265	BRCA	0.452	FACETS	109	26.7312
SMC_266	BRCA	0.160	Sequenza	60	14.32029
SMC_267	BRCA	0.745	FACETS	83	19.09372
SMC_268	BRCA	0.250	Sequenza	99	32.45932
SMC_269	BRCA	0.220	Sequenza	22	7.637487
SMC_270	BRCA	0.130	Sequenza	18	12.41092
SMC_271	BRCA	0.320	Sequenza	184	18.13903
SMC_272	BRCA	0.432	FACETS	76	18.13903
SMC_273	BRCA	0.120	Sequenza	92	12.41092
SMC_274	BRCA	0.270	FACETS	12	12.41092
SMC_275	BRCA	0.574	FACETS	80	12.41092
SMC_276	BRCA	0.215	FACETS	46	12.41092

SMC_277	BRCA	0.274	FACETS	117	31.50463
SMC_278	BRCA	0.234	FACETS	25	12.41092
SMC_279	BRCA	0.470	FACETS	47	12.41092
SMC_280	BRCA	0.120	Sequenza	16	13.71996
SMC_281	BRCA	0.215	FACETS	40	17.37861
SMC_282	BRCA	0.423	FACETS	239	24.69592
SMC_283	BRCA	0.493	FACETS	19	12.80529
SMC_284	BRCA	0.617	FACETS	168	17.37861
SMC_285	BRCA	0.200	Sequenza	104	14.63462
SMC_286	BRCA	0.049	Mutation-based	27	11.89063
SMC_287	BRCA	0.494	FACETS	68	10.97596
SMC_288	BRCA	0.685	FACETS	189	17.18435
SMC_289	BRCA	0.610	Sequenza	65	5.728115
SMC_290	BRCA	0.216	FACETS	28	10.50154
SMC_291	BRCA	0.366	FACETS	72	19.09372
SMC_292	BRCA	0.259	FACETS	75	18.29327
SMC_293	BRCA	0.809	FACETS	14	9.146637
SMC_294	BRCA	0.250	FACETS	37	12.80529
SMC_295	BRCA	0.330	PureCN	43	13.71996
SMC_296	BRCA	0.719	FACETS	95	14.63462
SMC_297	BRCA	0.333	FACETS	24	6.682801
SMC_298	BRCA	0.344	FACETS	55	8.592173
SMC_299	BRCA	0.098	Mutation-based	38	14.32029
SMC_300	BRCA	0.110	Sequenza	98	10.50154
SMC_301	BRCA	0.562	FACETS	77	14.63462
SMC_302	BRCA	0.017	Mutation-based	24	16.46395
SMC_303	BRCA	0.207	FACETS	52	8.231973
SMC_304	BRCA	0.489	FACETS	11	14.32029

SMC_305	BRCA	0.365	FACETS	23	15.27497
SMC_306	BRCA	0.420	FACETS	28	10.50154
SMC_307	BRCA	0.386	FACETS	37	10.50154
SMC_308	BRCA	0.382	FACETS	18	14.32029
SMC_309	BRCA	0.606	FACETS	47	17.18435
SMC_310	BRCA	0.549	FACETS	46	13.3656
SMC_311	BRCA	0.524	FACETS	99	15.27497
SMC_312	BRCA	0.413	FACETS	82	19.09372
SMC_313	BRCA	0.043	Mutation-based	28	12.41092
SMC_314	BRCA	0.445	FACETS	55	12.41092
SMC_315	BRCA	0.446	FACETS	169	18.29327
SMC_316	BRCA	0.130	Sequenza	127	10.97596
SMC_317	BRCA	0.695	FACETS	13	5.487982
SMC_318	BRCA	0.164	FACETS	16	12.80529
SMC_319	BRCA	0.567	FACETS	131	19.20794
SMC_320	BRCA	0.839	FACETS	34	8.231973
SMC_321	BRCA	0.215	FACETS	52	14.63462

BRCA, breast cancer; *NSCLC*, non-small-cell lung cancer; *pTMB*, panel sequencing-based tumor mutational burden; *wTMB*, whole-exome sequencing-based tumor mutational burden.

Table S3. TMB classification performance between WES-based TMB and panel-based TMB in the paired-NSCLC and paired-BRCA cohorts: Implications for the proportion of reclassified patients according to the tumor purity.

NSCLC (n =156)		Total Patients		Purity over 30%		Purity under 30%		BRCA (n = 165)		Total Patients		Purity over 30%		Purity under 30%	
		Panel-TMB		Panel-TMB		Panel-TMB				Panel-TMB		Panel-TMB		Panel-TMB	
		High	Low	High	Low	High	Low			High	Low	High	Low	High	Low
WES-TMB	High	20	11	13	9	7	2	WES-TMB	High	16	16	12	8	4	8
	Low	11	114	6	59	5	55		Low	11	122	5	58	6	64
**PPA(%)**		**64.52%**		**68.42%**		**58.33%**				**59.26%**		**70.59%**		**40.00%**	
**NPA(%)**		91.20%		86.76%		96.49%				88.41%		87.88%		88.89%	
**OPA(%)**		85.90%		82.76%		89.86%				83.64%		84.34%		82.93%	

BRCA, Breast cancer; *NPA*, negative percent agreement; *NSCLC*, non-small-cell lung cancer; *OPA*, overall percent agreement; *PFS*, progression-free survival; *PPA*, positive percent agreement; *TMB*, tumor mutational burden; *WES*, whole-exome sequencing.

Table S4. Sensitivity analyses of multivariable cox-regression for PFS of the Paired-NSCLC cohort with available PD-L1 expression level: Tumor purity-stratified analyses

		**Total Patients with available PD-L1 data (n = 111)**				**Adequate Purity (n = 63)**				**Low Purity (n = 48)**			
		**Model 1 (WES-TMB)**		**Model 2 (Panel-TMB)**		**Model 1 (WES-TMB)**		**Model 2 (Panel-TMB)**		**Model 1 (WES-TMB)**		**Model 2 (Panel-TMB)**	
Variables		aHR (95% CI)	*P*	aHR (95% CI)	*P*	aHR (95% CI)	P	aHR (95% CI)	*P*	aHR (95% CI)	*P*	aHR (95% CI)	*P*
Age	(cont)	1.00 (0.98–1.03)	0.762	1.00 (0.98–1.02)	0.804	0.99 (0.96–1.02)	0.484	0.99 (0.96–1.02)	0.526	1.00 (0.96–1.04)	0.904	1.01 (0.97–1.05)	0.59
Sex	M	1 (Reference)	-	1 (Reference)	-	1 (Reference)	-	1 (Reference)	-	1 (Reference)	-	1 (Reference)	-
	F	1.00 (0.62–1.62)	0.984	0.95 (0.59–1.54)	0.84	1.13 (0.60–2.15)	0.699	1.15 (0.61–2.18)	0.663	0.95 (0.39–2.29)	0.907	0.72 (0.30–1.76)	0.477
Lines of Therapy Received	0	1 (Reference)	-	1 (Reference)	-	1 (Reference)	-	1 (Reference)	-	1 (Reference)	-	1 (Reference)	-
	1	1.65 (0.63–4.33)	0.306	1.99 (0.75–5.24)	0.165	1.60 (0.44–5.77)	0.473	1.87 (0.52–6.71)	0.338	1.10 (0.21–5.68)	0.91	1.83 (0.33–10.07)	0.489
	2	1.24 (0.46–3.32)	0.67	1.47 (0.55–3.97)	0.444	1.90 (0.51–7.13)	0.343	2.52 (0.66–9.59)	0.176	0.61 (0.11–3.37)	0.573	0.95 (0.17–5.41)	0.952
	3	1.34 (0.50–3.65)	0.561	1.52 (0.56–4.14)	0.416	1.33 (0.36–4.89)	0.669	1.37 (0.38–4.98)	0.631	0.70 (0.11–4.35)	0.699	1.38 (0.21–9.09)	0.735
ECOG PS	0	1 (Reference)	-	1 (Reference)	-	1 (Reference)	-	1 (Reference)	-	1 (Reference)	-	1 (Reference)	-
	1	2.04 (0.27–15.39)	0.488	2.46 (0.33–18.56)	0.383	ND	0.996	ND	0.996	0.29 (0.03–2.81)	0.286	0.30 (0.03–2.91)	0.301
	2	2.74 (0.32–23.16)	0.355	3.67 (0.43–31.40)	0.235	ND	0.996	ND	0.996	0.59 (0.05–6.72)	0.672	0.66 (0.06–7.36)	0.735
PD-L1 Level	Low	1 (Reference)	-	1 (Reference)	-	1 (Reference)	-	1 (Reference)	-	1 (Reference)	-	1 (Reference)	-
	High	0.72 (0.46–1.14)	0.16	0.83 (0.52–1.32)	0.43	1.08 (0.57–2.06)	0.807	1.08 (0.56–2.06)	0.827	0.42 (0.19–0.91)	0.029	0.60 (0.26–1.37)	0.226
**TMB group**	Low	1 (Reference)	-	1 (Reference)	-	1 (Reference)	-	1 (Reference)	-	1 (Reference)	-	1 (Reference)	-
	**High**	**0.56 (0.32–0.98)**	**0.043**	**0.38 (0.20–0.71)**	**0.002**	**0.42 (0.20–0.89)**	**0.023**	**0.34 (0.14–0.79)**	**0.012**	**0.79 (0.29–2.19)**	**0.657**	**0.33 (0.11–1.05)**	**0.061**

aHR, adjusted hazard ratio; *CI*, confidence interval; *ND*, not determined; *NSCLC*, non-small-cell lung cancer; *PD-L1*, Programmed death-ligand 1; *PFS*, progression-free survival; *TMB*, tumor mutational burden; *WES*, whole-exome sequencing.

Table S5. List of clonal mutations used for the analysis of association between distribution of VAF and tumor purity, related to Figure 3.

TERT:Promoter Mutations	TP53:S215G	MET:V1206L	PIK3CA:R38H	PTEN:Y68D	PTEN:T131I	PTEN:T160I	KRAS:G12V
TERT:C228T	TP53:K120M	MET:X1007_splice	PIK3CA:Q546P	PTEN:L325F	PTEN:H93Q	PTEN:R130A	KRAS:E62K
TERT:C250T	TP53:Y220C	MET:M1250T	PIK3CA:P449T	PTEN:R335L	PTEN:R130*	PTEN:K128Q	KRAS:L19F
EGFR:exon19del	TP53:V218dup	MET:Y1003F	PIK3CA:Q546K	PTEN:D92E	PTEN:R130L	PTEN:V217D	KRAS:P34R
EGFR:T263P	TP53:V157D	MET:981_1028splice	PIK3CA:N345K	PTEN:C124N	PTEN:G165E	PTEN:A126G	KRAS:Q61R
EGFR:exon20ins	TP53:H179L	MET:Y1230H	PIK3CA:E542V	PTEN:D252G	PTEN:T131A	PTEN:F241S	KRAS:A11_G12insGA
EGFR:L858R	TP53:R175H	MET:V1088A	PIK3CA:H1047L	PTEN:N48K	PTEN:G36R	PTEN:V343E	KRAS:G13C
EGFR:L747P	TP53:G244S	MET:H1112Y	PIK3CA:T1025S	PTEN:G127N	PTEN:T167A	PTEN:K128R	KRAS:G12R
EGFR:C620Y	TP53:L145R	MET:H1112R	PIK3CA:E453K	PTEN:Y65C	PTEN:E157G	PTEN:M199del	KRAS:Q22K
EGFR:G735S	TP53:P151S	MET:F1218I	PIK3CA:Q546R	PTEN:D92V	PTEN:D92A	PTEN:G127E	KRAS:F156L
EGFR:A289V	TP53:E285V	MET:V1110I	PIK3CA:E545G	PTEN:H61D	PTEN:A126S	PTEN:H123Y	KRAS:Y64A
EGFR:G719S	TP53:R273H	MET:Y1235D	AKT2:E17K	PTEN:H93D	PTEN:G129R	PTEN:I122L	KRAS:G12C
EGFR:E709K	TP53:I254T	MET:X1006_splice	FGFR1:N546K	PTEN:G165V	PTEN:C71Y	PTEN:I168F	KRAS:Q61L
EGFR:A750P	SMAD4:D537Y	MET:Y1248C	APC:R640G	PTEN:D92G	PTEN:D162H	PTEN:A39P	KRAS:A18D
EGFR:G724S	FBXW7:R505L	MET:X1009_splice	IL7R:T244_I245insCPT	PTEN:F347L	PTEN:T131L	BRAF:L485_P490del	KRAS:Q22E
EGFR:L861Q	FBXW7:R465C	MET:X1008_splice	CCND1:P287T	PTEN:K128N	PTEN:G129E	BRAF:G469A	KRAS:K147E
EGFR:R324L	FBXW7:R479Q	MET:Y1253D	CCND1:T286A	PTEN:D92H	PTEN:H93R	BRAF:F468C	KRAS:K5N
EGFR:N826S	FBXW7:R505C	MET:L1213V	CCND1:D289del	PTEN:I122S	PTEN:A126V	BRAF:T599R	KRAS:F28L
EGFR:G598V	FBXW7:R482Q	MET:963_D1010splice	CCND1:T286I	PTEN:G129D	PTEN:L112P	BRAF:p61BRAF	KRAS:V14I
EGFR:R108K	RBM10:V354E	MET:F1200I	KEAP1:R272C	PTEN:R173H	PTEN:K125M	BRAF:V600E	KRAS:G10dup
EGFR:L861R	TSC2:H597Y	MET:X963_splice	KEAP1:G430C	PTEN:D24Y	PTEN:D326N	BRAF:V471F	KRAS:S65N
EGFR:E884K	TSC2:A889P	MET:M1268T	KEAP1:G423V	PTEN:L112R	PTEN:K125L	BRAF:V600R	KRAS:K117N
EGFR:A864T	TSC2:L410R	MET:N1118Y	KEAP1:S243C	PTEN:H118P	PTEN:A126D	BRAF:T599dup	KRAS:G12A
EGFR:L833V	TSC2:S1653F	PIK3CA:M1043V	KEAP1:R320Q	PTEN:H123Q	PTEN:Y155C	BRAF:L505H	KRAS:G13D
EGFR:E330K	TSC2:Y1571N	PIK3CA:E545A	KEAP1:G333S	PTEN:R130G	PTEN:S170R	BRAF:V600M	KRAS:G12S
EGFR:H773L	TSC2:T1623I	PIK3CA:Q546E	KEAP1:R470C	PTEN:K128T	PTEN:P95S	BRAF:T599_V600insV	KRAS:Q61H
EGFR:S768I	TSC2:V241del	PIK3CA:P539R	KEAP1:G186R	PTEN:N94I	PTEN:P96Q	BRAF:T599insTT	KRAS:P34L
EGFR:G719A	TSC2:S1653P	PIK3CA:E545K	KEAP1:G333C	PTEN:C124R	PTEN:V343L	BRAF:G464V	KRAS:A59G
CHEK2:K373E	TSC2:L792R	PIK3CA:K111E	KEAP1:V155F	PTEN:D162G	PTEN:H93Y	BRAF:V600K	KRAS:G60R

TP53:N235D	TSC2:V705E	PIK3CA:E542K	KEAP1:R415G	PTEN:F341V	PTEN:P95L	BRAF:V600D_K601insFGLAT	KRAS:A146T
TP53:G245S	TSC2:H1620R	PIK3CA:H1047R	KEAP1:D422N	PTEN:L23F	PTEN:M35R	BRAF:L597V	KRAS:T58I
TP53:R249W	TSC2:L830R	PIK3CA:N1044K	PTEN:K125E	PTEN:C136Y	PTEN:A34D	BRAF:V600D	KRAS:D153V
TP53:G244R	TSC2:E1552del	PIK3CA:E545Q	PTEN:H123D	PTEN:R15S	PTEN:P169H	BRAF:V600delinsYM	KRAS:Y71H
TP53:R280K	TSC2:T1203K	PIK3CA:C420R	PTEN:K62R	PTEN:R130K	PTEN:G44D	BRAF:V600_K601delinsE	
TP53:K120E	MET:Y1248H	PIK3CA:M1043I	PTEN:F154L	PTEN:R161G	PTEN:R335*	NOTCH1:L1574P	
TP53:N239S	MET:H1112L	PIK3CA:H1047Y	PTEN:A121E	PTEN:L181P	PTEN:R15K	KRAS:G12D	

VAF, variant allele frequency.
