# Supplementary material for: Clinical advantage of targeted sequencing for unbiased tumor mutational burden estimation in samples with low tumor purity
Source: J Immunother Cancer. 2020 Oct 19;8(2):e001199. doi: 10.1136/jitc-2020-001199 (PMC7574938; doi:10.1136/jitc-2020-001199)
Supplement: Supplementary data [file jitc-2020-001199supp003.pdf]

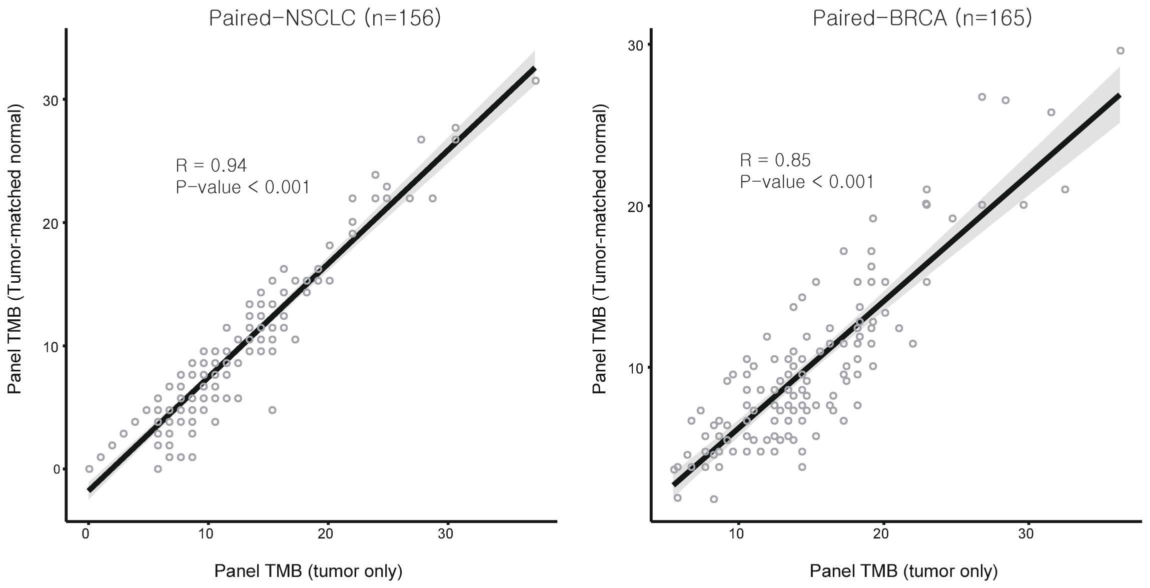

Supplementary Fig. S1. Strong correlation between the two strategies of panel-based TMB (tumor only vs. matched normal) found in the paired NGS cohorts. (a) Paired-NSCLC cohort (n = 156). (b) Paired-BRCA cohort (n = 165). *BRCA*, breast cancer; *NGS*, next-generation sequencing; *NSCLC*, non-small-cell lung cancer; *TMB*, tumor mutational burden.

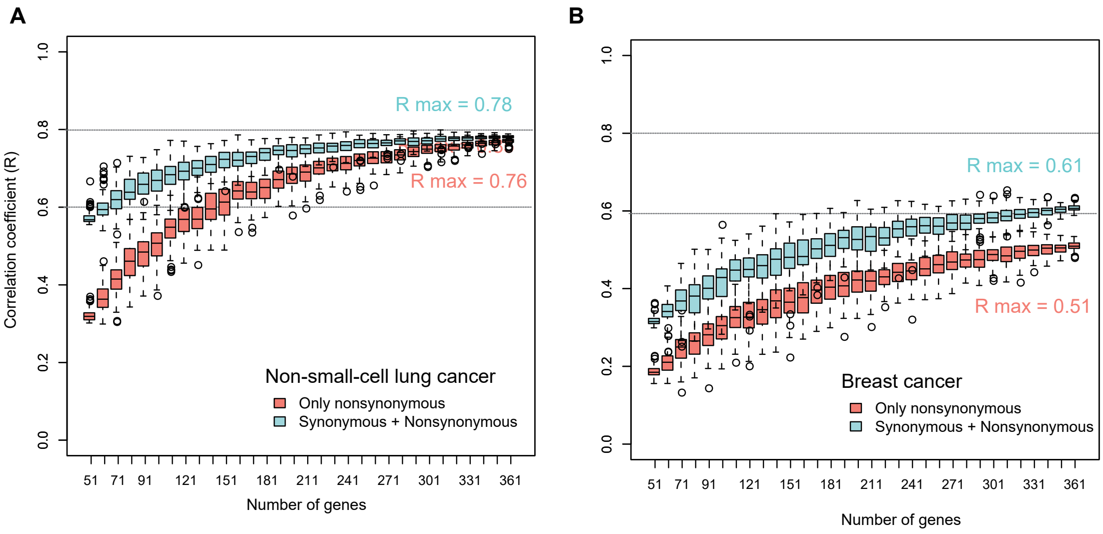

Supplementary Fig. S2. Correlation coefficients between the two TMB estimates (WES-based TMB and panel-based TMB) according to the number of included genes. The impact of synonymous alterations in panel-based TMB was also analyzed. (a) Paired-NSCLC cohort. (b) Paired-BRCA cohort. *BRCA*, breast cancer; *NGS*, next-generation sequencing; *NSCLC*, non-small-cell lung cancer; *TMB*, tumor mutational burden; *WES*, whole-exome sequencing.

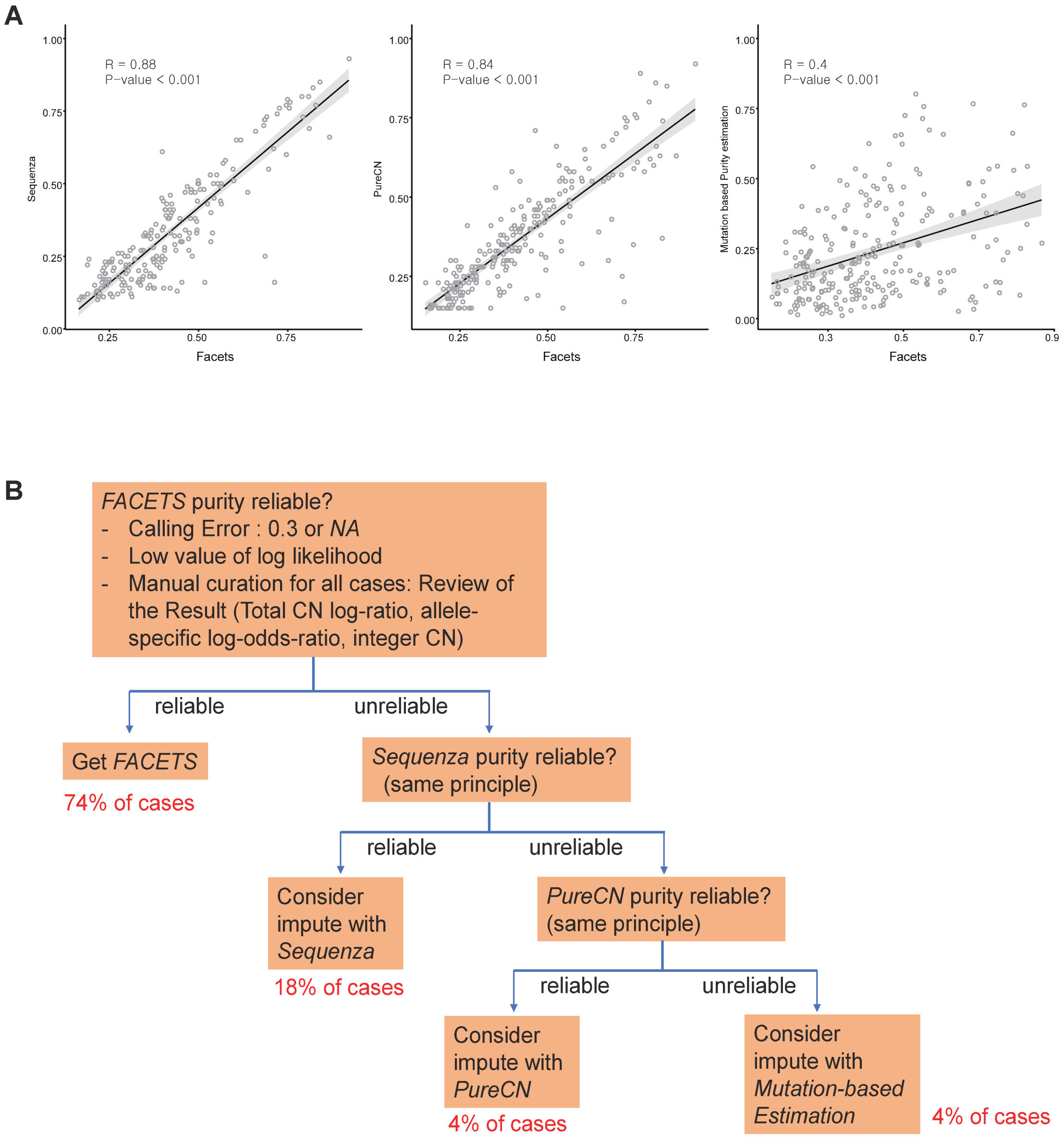

Supplementary Fig. S3. Correlations between computational algorithms for tumor purity and ploidy estimation, and a decision tree to determine tumor purity. (a) Correlations of three methods (Sequenza, PureCN, and mutation-based estimation) against FACETS. (b) Decision tree for determining tumor purity in paired NGS cohorts of this study. *CN*, copy number; *NA*, not applicable; *NGS*, next-generation sequencing.

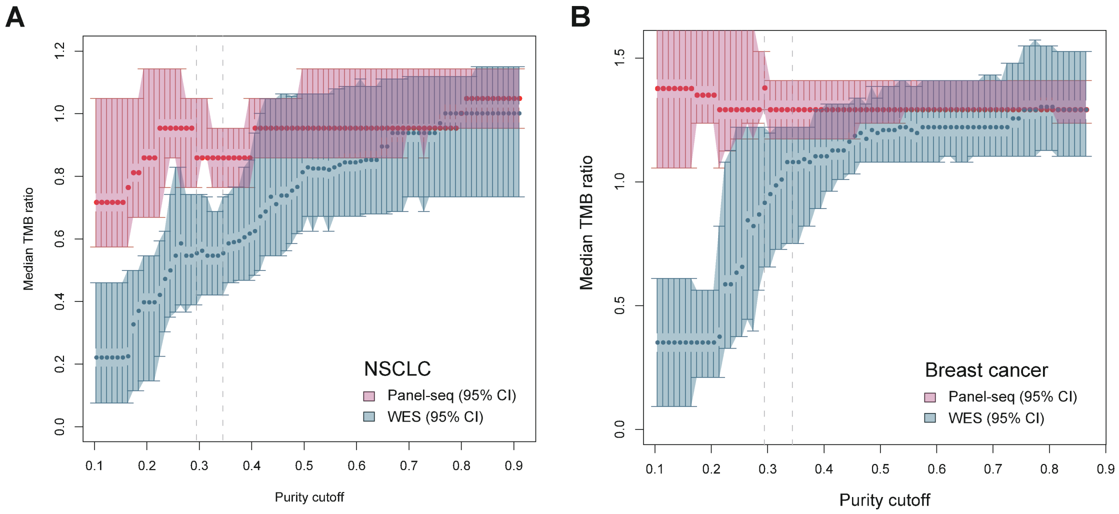

Supplementary Fig. S4. Cut-off point analysis to determine low tumor purity with the degree of underestimation in the two TMB estimates (WES-based TMB and panel-based TMB). (a) Paired-NSCLC cohort. (b) Paired-BRCA cohort. *BRCA*, breast cancer; *CI*, confidence interval; *NSCLC*, non-small-cell lung cancer; *TMB*, tumor mutational burden; *WES*, whole-exome sequencing.

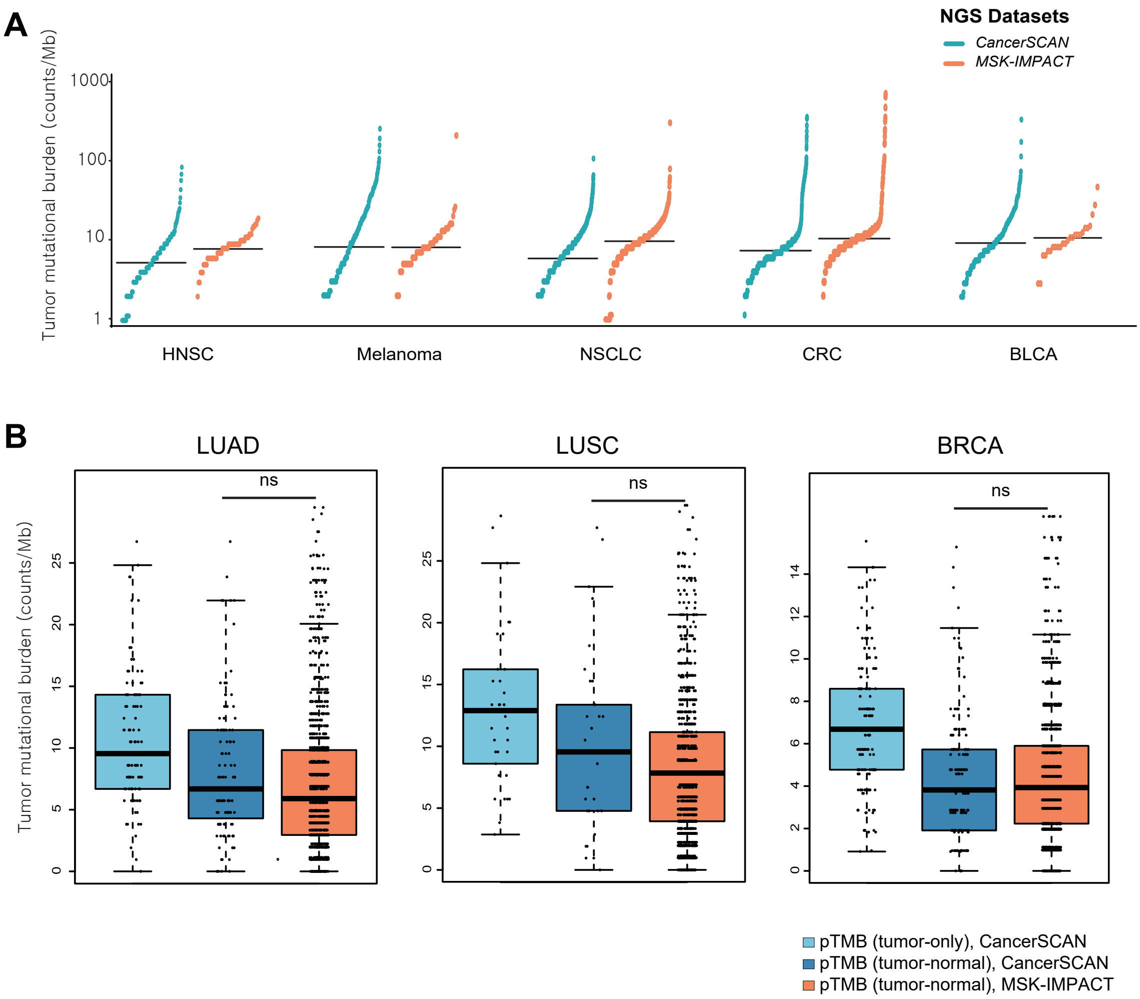

Supplementary Fig. S5. Distribution of pTMB from the two NGS datasets (CancerSCAN and MSK-IMPACT) and the impact of germline sequencing on the pTMB estimates. (a) Comparison of two institutional pTMB estimates across five tumor types associated with the benefit of ICI (HNSC, Melanoma, NSCLC, CRC, and BLCA). (b) Impact of germline sequencing on the pTMB estimates in paired-NSCLC and paired-BRCA cohorts. *BLCA,* bladder cancer; *BRCA*, breast cancer; *CRC*, colorectal cancer; *HNSC*, head and neck squamous cell carcinoma; *ICI*, immune checkpoint inhibitor; *NGS*, next-generation sequencing; *NSCLC*, non-small cell lung cancer; *pTMB*, panel sequencing-based tumor mutational burden.

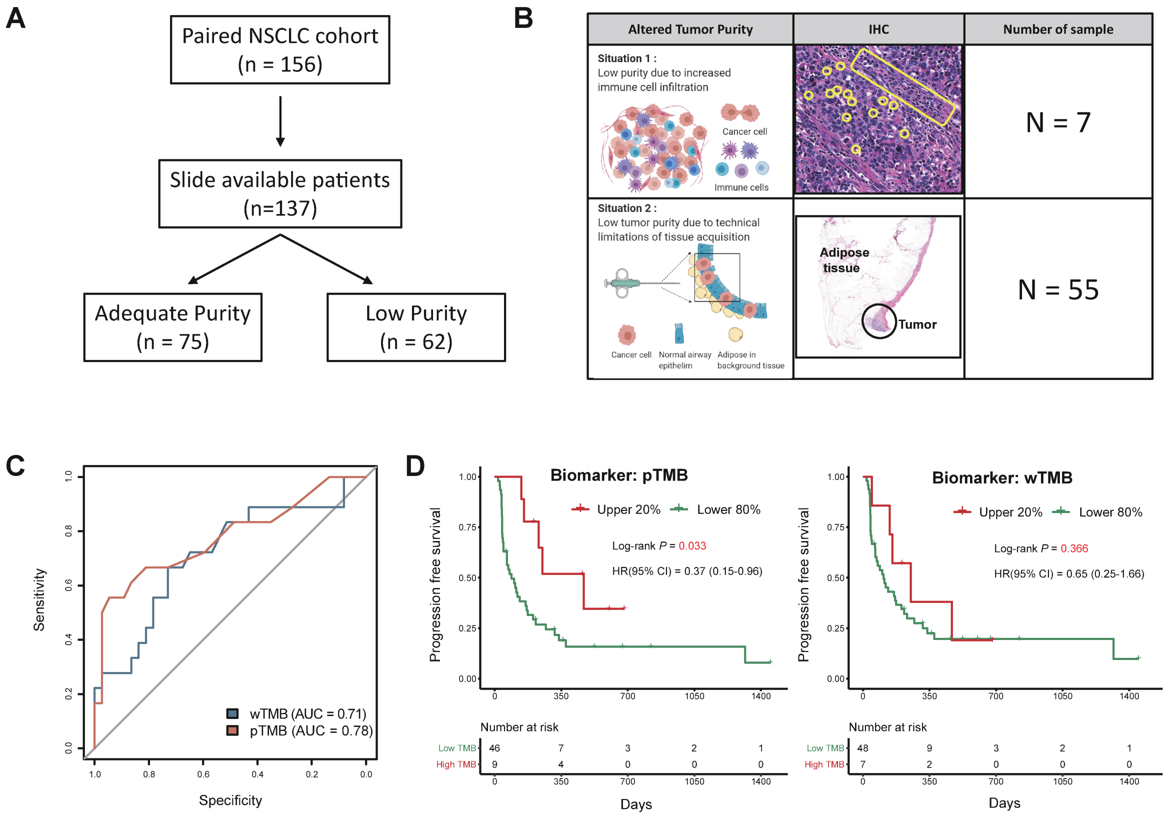

Supplementary Fig. S6. The performance of pTMB and wTMB in the low tumor purity, in the setting of low- or no infiltration of immune cell infiltration. (a) Patients with available H&E sections. (b) Two representative situations related to low tumor purity. (c) AUC comparison of pTMB with wTMB in terms of predicting response. (d) Survival stratification with the use of pTMB and wTMB. AUC, area under the curve; IHC, immunohistochemistry; wTMB, whole-exome sequencing-based tumor mutational burden; pTMB, panel sequencing-based tumor mutational burden.

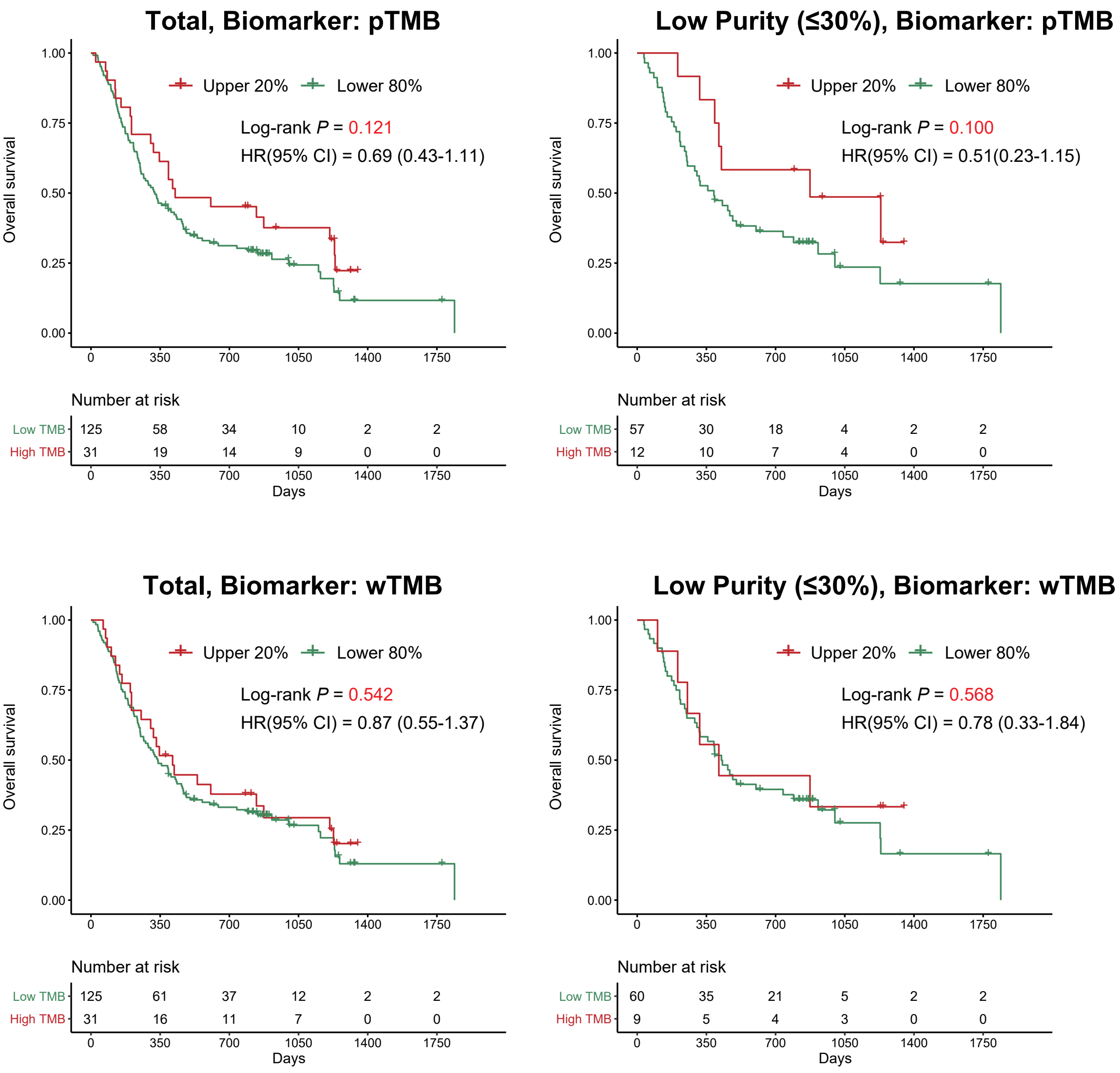

Supplementary Fig. S7. Overall survival analysis of pairedNSCLC cohort with the use of pTMB (toppanel) and the use of wTMB (bottom panel). wTMB, whole exome sequencing-based tumor mutational burden; pTMB, panel sequencing-based tumor mutational burden.

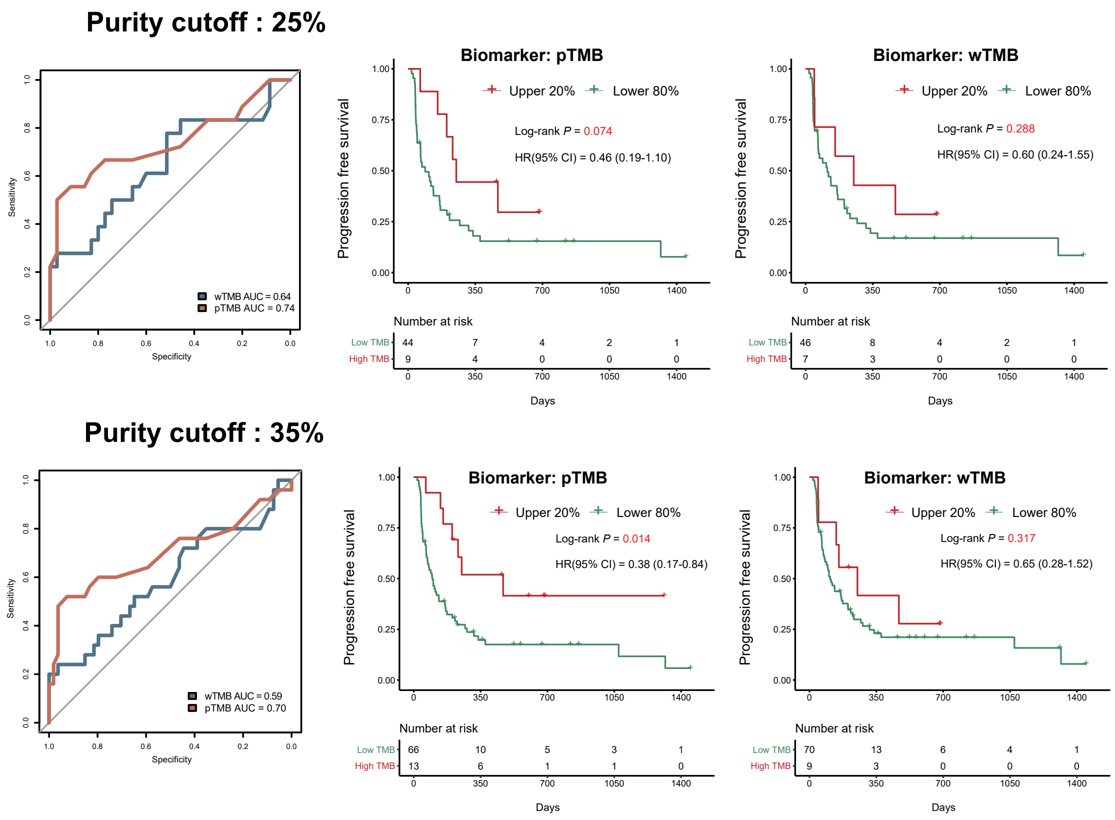

Supplementary Fig. S8. Sensitivity analyses of PFS in the paired NSCLC cohort with various definitions of tumor purity cutoff (25% and 35%). NSCLC, non small cell lung cancer; PFS, progression -free survival.

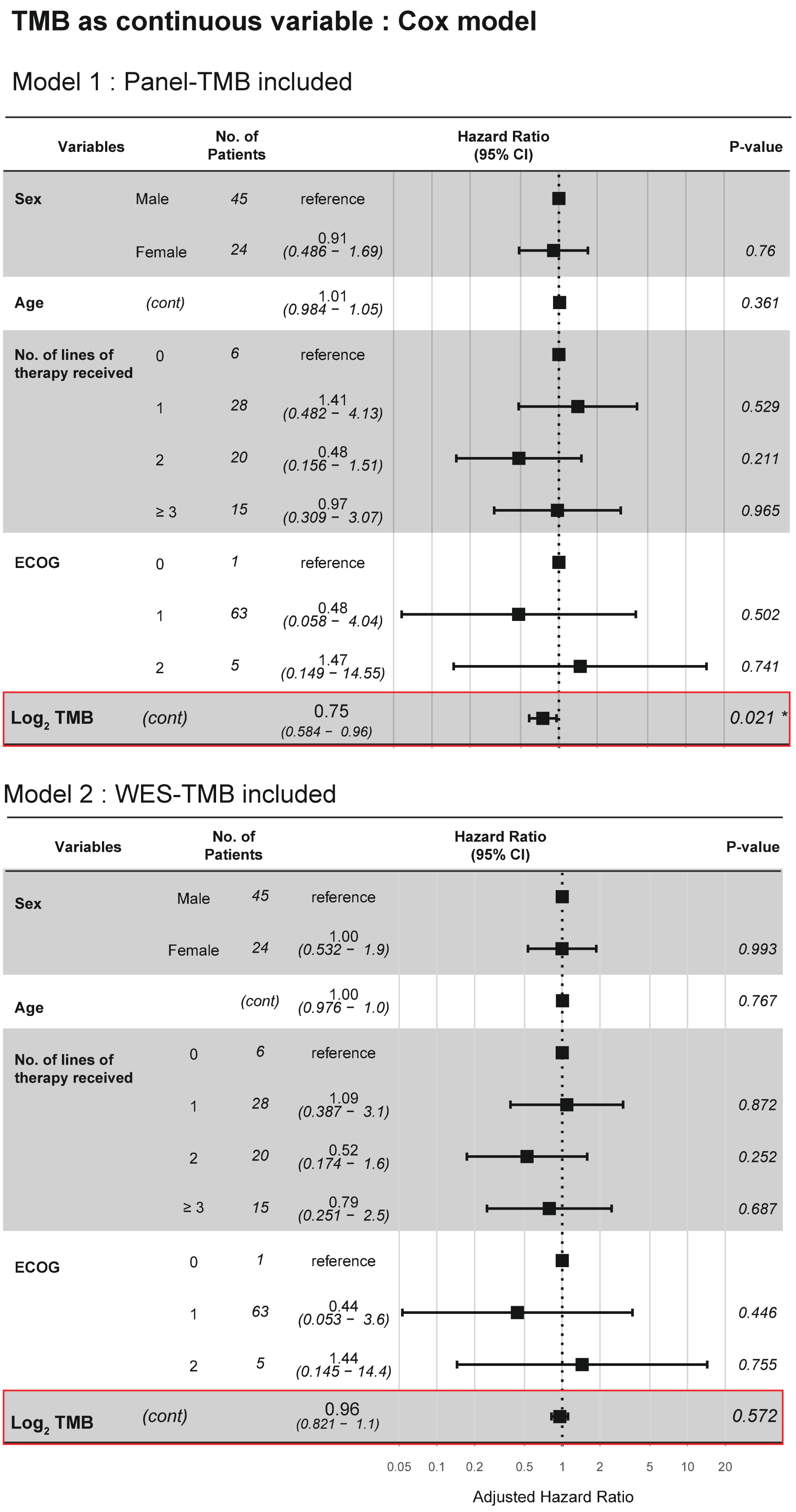

Supplementary Fig. S9. Multivariable cox regression models of PFS incorporating TMB as a continuous variable. Model1: pTMB (log transformed), and model 2: wTMB (log transformed). wTMB, whole exome sequencing based tumor mutational burden; pTMB, panel sequencing-based tumor mutational burden.
